# Two Worlds, One Battle: How Bacteria and Malignancies Converge on Drug Resistance

**DOI:** 10.3390/ijms27104239

**Published:** 2026-05-10

**Authors:** Christos Papaneophytou

**Affiliations:** Department of Life Sciences, School of Life and Health Sciences, University of Nicosia, Nicosia 2417, Cyprus; papaneophytou.c@unic.ac.cy; Tel.: +357-22841941

**Keywords:** drug resistance, antimicrobial resistance, cancer drug resistance, efflux pumps, metabolic reprogramming, persister cells, combination therapy, drug repurposing

## Abstract

Drug resistance represents one of the most critical challenges in modern medicine, undermining the efficacy of therapies across both bacterial infections and cancer. Although these conditions arise in fundamentally distinct biological systems, they are governed by shared evolutionary pressures that drive the emergence and selection of resistant populations. This narrative review provides an integrative, cross-disciplinary perspective on drug resistance, focusing on bacteria and cancer and emphasizing the shared evolutionary and molecular mechanisms underlying treatment failure in both domains. Key resistance strategies include efflux-mediated drug export, target modification, enzymatic drug inactivation, metabolic reprogramming, epigenetic and transcriptional plasticity, and protection conferred by specialized microenvironments. These processes are further reinforced by phenotypic heterogeneity, including bacterial persister cells and cancer stem-like cells, which contribute to recurrence and multidrug resistance. Collectively, these parallels define drug resistance as a convergent evolutionary phenomenon driven by adaptability under selective pressure. Recognizing these shared mechanisms reveals important translational opportunities for therapeutic intervention. Strategies such as combination therapy, drug repurposing, nanotechnology-enabled delivery systems, and host-directed approaches offer promising avenues to prevent, delay, or overcome resistance. By integrating insights from microbiology and oncology, this review proposes a unified framework for resistance biology and highlights the potential of cross-disciplinary strategies to improve treatment durability and clinical outcomes.

## 1. Introduction

Drug resistance refers to the reduced effectiveness of a treatment against a disease or condition [1]. It most commonly arises in microorganisms and cancer cells, which can survive treatments that were previously effective, posing a major threat to global health [2]. Driven mainly by evolutionary pressure for survival, the emergence of resistance is one of the biggest challenges in modern medicine. It weakens the effectiveness of current therapies, limits treatment choices, and is prevalent in both infectious diseases and cancer [3]. Essentially, any biological system capable of evolving and producing phenotypic variation can develop resistance under selective pressure, whether this variation pre-exists within a population or appears newly after exposure to a therapeutic agent [4]. At the molecular and population levels, the same core evolutionary principles, including clonal selection, fitness landscape remodeling, epistatic interactions, and phenotypic plasticity, operate across microbial and neoplastic systems, suggesting that resistance is best understood as a general property of adapting cell populations rather than a phenomenon unique to any single disease context [5].

Therapeutic resistance has long been a persistent challenge that continues to develop. Each new treatment applies selective pressure, quickly favoring resistant subpopulations. This recurring pattern, while a serious clinical concern, also provides valuable insight into the mechanisms of resistance and strategies that may limit, redirect, or even reverse its development [6]. In cancer, inherent genomic instability and ongoing therapeutic stress lead to changes in the expression, mutation status, and copy number of numerous genes across multiple signaling networks, allowing tumor cells to survive despite targeted therapies. Both driver and non-driver gene alterations can affect drug sensitivity and create resistance [7]. Similarly, in microbial infections, processes such as mutation under selective pressure, horizontal gene transfer (HGT), and phenotypic switching drive the continuous emergence and spread of resistant strains [8].

The clinical importance of drug resistance became particularly clear in cases of human immunodeficiency virus 1 (HIV-1) infection. The introduction of combination antiretroviral therapy in the mid to late 1990s, which used multiple direct-acting antivirals targeting different steps in the viral life cycle, greatly reduced viral load and improved patient outcomes [9]. A decade after the success of combination antiviral therapy for HIV, similar multi-drug, direct-acting antiviral strategies led to the development of curative treatments for hepatitis C virus (HCV) infection, with modern direct-acting antiviral (DAA) combinations achieving sustained virologic response rates above 90% [10]. Over the last twenty years, these approaches have been increasingly used in oncology, where research and pharmaceutical innovation have driven major advances in targeted and combination therapies. However, treatment failure due to resistance still remains common [11]. While these developments showed that mechanism-based combination therapy can suppress resistance, they also revealed an important disparity: the success of combination strategies in specific viral cases has not broadly translated to bacterial, fungal, or parasitic infections, partly because biological features such as HGT, environmental reservoirs, and polymicrobial complexity pose unique challenges. As a result, antimicrobial resistance (AMR) remains widespread and continues to grow across various infectious pathogens [12].

AMR, broadly defined as the ability of bacteria, viruses, fungi, and parasites to develop mechanisms that confer resistance to antimicrobial agents, is one of the most urgent global health threats of the 21st century [13]. Resistance has now been documented across all major antibiotic classes in clinical use, while the pipeline for developing new antimicrobials remains critically limited. Awareness of AMR as a measurable public health burden has increased over the past two decades, driven by initiatives like the World Health Organization’s 2001 Global Strategy on AMR and regional surveillance networks, including the European Antimicrobial Resistance Surveillance Network (EARS-Net), which have facilitated systematic estimates of resistance-related illness and death [14].

Recent analyses underscore the severity of the AMR crisis, with a global study linking over one million deaths each year to drug-resistant bacteria like *Escherichia coli* and *Staphylococcus aureus* [15]. In 2021 alone, antimicrobial-resistant infections were estimated to have caused about 1.14 million deaths worldwide, a number projected to increase to 1.91 million by 2050 [16]. A 2014 UK Government review projected that, without coordinated global action, AMR could cause up to 10 million deaths annually and $100 trillion (USD) in economic losses by 2050. These projections are based on scenario modeling and limited data, so they should be seen as indicators of the potential scale of the crisis rather than precise predictions [17]. According to the Global Antimicrobial Resistance and Use Surveillance System (GLASS) [18], roughly 35% of *S. aureus* isolates worldwide are methicillin-resistant, while 42% of *E. coli* isolates are resistant to third-generation cephalosporins, significantly limiting treatment options [19]. In the United States, the Centers for Disease Control and Prevention estimates that over 2.8 million antibiotic-resistant infections occur each year, resulting in around 35,000 deaths [20]. The economic impact is also significant: AMR-related healthcare costs surpass € 9 billion annually in Europe and nearly $20 billion (USD) in the US, with an additional $35 billion (USD) in productivity losses (reviewed in [21]). Although resistance is a natural consequence of evolution, its rapid spread is accelerated by widespread inappropriate antimicrobial use, poor infection control, and efficient horizontal transfer of resistance genes among microbial populations [2,22].

Drug resistance is equally important in oncology. Cancer drug resistance is a common and clinically significant issue, especially in patients with advanced or metastatic disease. Despite notable progress in cancer treatments, including targeted therapies, immunotherapies, and precision medicine strategies, resistance often develops and causes disease progression and treatment failure [23]. It is estimated that resistance causes about 90% of cancer-related deaths, although this widely cited figure originates from early studies, and the exact percentage varies depending on cancer type and stage [24,25]. The patterns of resistance are well documented across different tumor types. In non-small cell lung cancer, acquired resistance to EGFR tyrosine kinase inhibitors often occurs through the T790M gatekeeper mutation [26]. In melanoma, resistance to BRAF inhibitors develops via reactivation of the MAPK pathway or alternative kinase signaling [27]. In ovarian cancer, platinum resistance arises through increased DNA damage repair, drug efflux, and changes in apoptotic pathways [28]. These examples highlight a common principle shared with microbial systems: resistance usually develops through a limited set of conserved adaptive strategies, such as target modification, drug efflux, pathway rewiring, and survival signaling, which recur across different biological contexts [29].

Despite these parallel challenges, progress in tackling drug resistance has been inconsistent across fields. Oncology drug development has gained from sustained pharmaceutical investment, fueled by precision medicine markets, supportive regulatory conditions, and high returns on innovation [30]. Conversely, antibiotic development continues to struggle with market failure, driven by low profitability, short treatment durations, and stewardship restrictions that discourage industry involvement. Between 2003 and 2013, more than two-thirds of major pharmaceutical companies withdrew from antibiotic research and development, reducing available treatment options as resistance rose [31].

The converging biological, clinical, and economic pressures related to therapeutic resistance highlight the urgent need for integrated strategies. Although resistance has been widely studied in infectious diseases and cancer, these fields have developed largely in isolation, with limited cross-disciplinary collaboration. However, the mechanistic and evolutionary similarities discussed earlier suggest that resistance in these systems is governed by common principles. Recognizing these similarities offers an opportunity to move beyond discipline-specific views, especially because approach-based strategies, including combination therapy, adaptive treatment planning, and biomarker-guided interventions, have already proven effective in certain settings and could be more widely applied when informed by insights from multiple fields.

Several previous reviews have addressed important aspects of this topic, including the evolutionary dynamics of resistance, the molecular basis of antimicrobial resistance, mechanisms of cancer drug resistance, and conceptual analogies between bacterial and malignant cell populations [32,33]. However, these reviews have generally focused on either a single biological domain, a limited set of mechanisms, or broad evolutionary principles, without systematically integrating mechanistic comparisons with translational and clinical implications. This review addresses this gap by providing a structured cross-disciplinary framework that directly compares bacterial infections and cancer across multiple levels: shared molecular mechanisms, adaptive cellular states, microenvironmental protection, therapeutic counterstrategies, limitations of cross-domain translation, and future research and policy priorities. Specifically, it examines genetic and epigenetic adaptation, efflux-mediated drug export, target modification, drug inactivation, metabolic reprogramming, cellular dormancy, and microenvironmental protection as convergent resistance strategies. It also discusses emerging therapeutic approaches, including combination therapy, drug repurposing, nanotechnology-enabled delivery systems, and host-directed interventions. By directly connecting concepts from microbiology and oncology, this review advances a unified framework for understanding resistance biology and highlights opportunities to improve treatment durability across clinical settings.

## 2. Drug Resistance Across Microbes and Cancer: A Brief Overview

Drug resistance is a key factor in treatment failure in two main areas of medicine: microbial infections and cancer. Despite their different origins, environments, and cellular structures, both microbes and cancer cells share the ability to adapt and survive under ongoing drug pressure. This section examines AMR and cancer drug resistance, focusing on their causes, clinical effects, and mechanisms.

### 2.1. When Bacteria Fight Back: The Rise in Antimicrobial Resistance

As discussed above, AMR refers to the ability of microorganisms (including bacteria, fungi, viruses, and parasites) to withstand the effects of drugs that were once effective against them [2,13]. This phenomenon represents a global health crisis caused mainly by the overuse and misuse of antimicrobial agents, which exert strong selection pressure promoting the development and spread of resistant strains [34]. The mechanisms underlying bacterial resistance are numerous and can be categorized into intrinsic, acquired, and adaptive forms, each with distinct genetic and phenotypic characteristics (Table 1).

Intrinsic resistance refers to a species-wide trait that is independent of prior antibiotic exposure and not attributable to HGT. In some cases, this resistance is constitutively expressed, whereas in others it may be inducible, with naturally encoded genes being upregulated to resistance-conferring levels only after exposure to specific antibiotics. By contrast, acquired resistance arises in previously susceptible bacteria through chromosomal mutation or the acquisition of exogenous genetic material, while adaptive resistance reflects a transient and often reversible response to environmental or antibiotic stress [35,36,37].

**Table 1:** Molecular Mechanisms of Antimicrobial Resistance ^1^.

Category	Mechanism	Molecular Basis	Representative Examples	Associated Pathogens	Affected Antibiotics
Intrinsic	Structural barriers	Outer membrane exclusion, lack of target	LPS barrier (Gram-negative); Absent PBPs (*Mycoplasma*)	Gram-negative bacteria, *Mycoplasma* spp.	Vancomycin, β-lactams
Intrinsic	Chromosomally encoded multidrug efflux pumps	Chromosomally encoded pumps	AcrAB-TolC, MexAB-OprM	*E. coli*, *P. aeruginosa*	Multiple classes
Acquired	Enzymatic inactivation	β-lactamases, aminoglycoside-modifying enzymes	ESBLs, KPC, NDM-1, AAC enzymes	*K. pneumoniae*, *E. coli*, *Acinetobacter*	β-lactams, aminoglycosides
Acquired	Target modification	PBP alterations, ribosomal methylation, and DNA gyrase mutations	PBP2a, VanA, *gyrA*/*parC* mutations	MRSA, VRE, fluoroquinolone-resistant Gram-negatives	β-lactams, glycopeptides, fluoroquinolones
Acquired	Mutation- or plasmid-associated overexpression of efflux systems	Plasmid or mutation-driven overexpression	TetA, MexXY, NorA	*S. aureus*, *P. aeruginosa*	Tetracyclines, fluoroquinolones
Acquired	Permeability reduction	Porin loss or modification	OmpK35/36 deletion, OprD loss	CRE, *P. aeruginosa*	Carbapenems
Adaptive	Biofilm formation	Extracellular matrix production, persister cells	Polysaccharide synthesis; eDNA	*P. aeruginosa*, *S. aureus*	Multiple classes/reduced susceptibility across diverse agents
Cross- cutting	Transfer of resistance determinants via plasmids, transposons, and integrons	Plasmids, transposons, integrons	Conjugative plasmids carrying resistance cassettes	Pan-bacterial	Multiple classes

^1^ Data was obtained from [38,39,40]. Abbreviations: AAC, aminoglycoside acetyltransferase; CRE, carbapenem-resistant Enterobacterales; eDNA, environmental DNA; ESBL, extended-spectrum β-lactamase; KPC, *Klebsiella pneumoniae* carbapenemase; LPS, lipopolysaccharide; MRSA, methicillin-resistant *Staphylococcus aureus*; NDM, New Delhi metallo-β-lactamase; PBP, penicillin-binding protein; VRE, vancomycin-resistant *Enterococcus.*

The mechanisms underlying intrinsic resistance are structurally and biochemically varied. They include the absence or structural modification of antimicrobial targets; for instance, the lack of canonical penicillin-binding proteins in *Mycoplasma* spp. confers natural resistance to β-lactam antibiotics [41]. Physical barriers also contribute significantly: the outer membrane of Gram-negative bacteria restricts the entry of large hydrophilic molecules, conferring innate resistance to glycopeptides such as vancomycin [42]. Additionally, chromosomally encoded efflux pumps and the constitutive production of drug-inactivating enzymes, such as AmpC β-lactamases in *Pseudomonas aeruginosa*, further enhance baseline resistance levels [43]. The intrinsic resistome encompasses these structural and enzymatic determinants, along with interconnected metabolic pathways, regulatory circuits, and stress-response systems that collectively define the baseline antimicrobial susceptibility profile of a bacterial species [33]. Clinically, intrinsic resistance determines which antibiotics are predictably ineffective against a given pathogen and must therefore be considered when designing empiric antimicrobial therapy [44].

Acquired resistance is an evolutionary process in which bacteria that were once susceptible develop resistance through chromosomal mutations or by acquiring external genetic material via HGT [21]. The three main mechanisms of HGT (transformation, transduction, and conjugation) facilitate the rapid spread of resistance genes within and between bacterial species. Acquired resistance is most often transmitted through plasmids gained via conjugation and can be either temporary or permanently inherited [45,46]. At the molecular level, acquired resistance is driven by several key mechanisms: enzymatic inactivation of antibiotics (for example, β-lactamases that hydrolyze penicillins and cephalosporins), modification of drug target sites (such as altered penicillin-binding proteins or ribosomal methylation), upregulation of efflux pump systems that actively export drugs from the cell, and reduced membrane permeability that limits intracellular drug buildup [47]. The rapid spread of resistance genes through HGT has resulted in the emergence of multidrug-resistant (MDR) and extensively drug-resistant (XDR) pathogens, significantly complicating infection control and reducing therapeutic effectiveness [45,48].

Adaptive resistance is a transient, environmentally contingent phenotype that arises in response to subinhibitory concentrations of antibiotics and specific environmental cues, including nutrient availability, pH, ionic concentrations, and other stress signals [49]. Unlike intrinsic and acquired resistance, adaptive resistance is typically reversible and reverts to the baseline phenotype upon removal of the inducing stimulus [50]. The biological mechanisms underlying adaptive resistance remain incompletely understood; however, they are thought to involve high mutation rates, gene amplification, transient upregulation of efflux pumps, biofilm formation, epigenetic modifications, and phenotypic heterogeneity within bacterial populations. Biofilm-associated bacteria, in particular, exhibit markedly reduced susceptibility to antimicrobials due to restricted drug penetration, altered metabolic states, and the presence of persister cells that tolerate antibiotic exposure [51].

The clinical consequences of antimicrobial resistance are profound. Resistant pathogens such as extended-spectrum β-lactamase (ESBL)-producing *E. coli*, methicillin-resistant *S. aureus* (MRSA), carbapenem-resistant Enterobacterales (CRE), and multidrug-resistant *P. aeruginosa* are associated with severe infections, prolonged hospital stays, higher complication rates, and increased mortality [52,53]. In immunocompromised populations, including patients with hematological malignancies or those undergoing intensive chemotherapy, AMR significantly undermines treatment outcomes and elevates the risk of fatal infections [53].

The rapid global spread of AMR is driven by antibiotic overuse and misuse in both human medicine and agriculture, inadequate infection prevention and control measures, environmental contamination, and gaps in antimicrobial stewardship and surveillance infrastructure [38,54]. The limited pipeline for novel antimicrobials, coupled with evolving resistance patterns and insufficient investment in diagnostics and alternative therapies, further complicates clinical management. Addressing AMR requires coordinated multidisciplinary approaches, including the One Health framework, which recognizes the interconnectedness of human, animal, and environmental health in the emergence and dissemination of resistance [55].

While the mechanisms described above have been characterized primarily in the context of microbial pathogens, many of the underlying principles (including genetic and epigenetic adaptation, active drug efflux, target modification, and protection conferred by specialized microenvironments) are not unique to bacteria. Cancer cells, despite their eukaryotic complexity and distinct evolutionary context, employ strikingly analogous strategies to evade chemotherapeutic pressure. The following section examines the mechanistic landscape of cancer drug resistance, highlighting both the parallels and distinctions that inform our understanding of therapeutic failure across these two biological systems.

### 2.2. When Tumors Outsmart Therapy: The Challenge of Cancer Drug Resistance

Cancer represents a substantial and growing global health challenge. According to the latest WHO/IARC estimates, approximately 20 million new cancer cases and 9.7 million cancer-related deaths occurred worldwide in 2022. Global cancer incidence is projected to continue rising over the coming decades, with substantial increases expected by 2050 due to population aging and growth [56]. A significant factor limiting therapeutic success is drug resistance, which remains one of the foremost challenges in oncology and is estimated to account for roughly 90% of cancer-related deaths [25]. Despite remarkable advances in chemotherapy, targeted therapies, immunotherapies, and precision medicine, most cancers eventually develop mechanisms that enable them to evade or neutralize therapeutic pressure [57].

Cancer drug resistance may be intrinsic, arising from pre-existing features in treatment-naive tumors, or acquired, emerging during or after therapy in response to selective pressure. Although many tumors initially respond to chemotherapy or targeted agents, they often develop resistance over time, leading to disease progression and increasingly aggressive, treatment-refractory phenotypes [58,59]. This dynamic adaptability underscores the need to understand resistance as a complex evolutionary process rather than a static phenotype.

Intrinsic resistance refers to the pre-existing, inherent insensitivity of tumor cells to anticancer therapies before any treatment exposure. This form of resistance is driven by genetic, epigenetic, phenotypic, and microenvironmental factors that render standard therapies ineffective from the outset [60,61]. Key contributors include constitutive activation of survival pathways such as PI3K/Akt/mTOR, RAS/RAF/MAPK, and EGFR; baseline overexpression of ATP-binding cassette (ABC) efflux transporters such as P-glycoprotein (P-gp) and breast cancer resistance protein (BCRP); defects in apoptotic machinery; and the presence of cancer stem cells (CSCs), which possess robust self-renewal and DNA repair capabilities [62]. Epigenetic modifications and transcriptional reprogramming further reinforce intrinsic resistance by establishing gene expression programs that promote drug insensitivity, while features of the tumor microenvironment (TME), including hypoxia, immune suppression, extracellular matrix barriers, and stromal protection, contribute to baseline resistance before therapy initiation [63]. Clinically, intrinsic resistance presents as primary treatment failure, where patients fail to respond to first-line therapies. Examples include resistance to EGFR inhibitors in non-small cell lung cancer or anti-EGFR antibodies in colorectal cancer due to pre-existing alterations such as KRAS or BRAF mutations or oncogenic fusions. Identifying intrinsic resistance mechanisms through molecular profiling is therefore essential for optimizing personalized therapy selection and designing rational combinatorial strategies that preempt resistance [64]. Acquired resistance develops during or after therapeutic exposure as tumor cells undergo genetic, epigenetic, metabolic, and microenvironmental adaptations that collectively rewire survival pathways [65]. The main mechanisms of cancer drug resistance are summarized in Table 2.

At the genetic level, mutations may alter the structure or function of the drug target, rendering targeted agents ineffective. Epigenetic modifications can reshape transcriptional landscapes to support drug tolerance, while treatment-induced upregulation of ABC transporters reduces intracellular drug accumulation through active efflux [66,75]. Metabolic reprogramming promotes detoxification or sequestration of therapeutic agents, enhanced DNA repair capacity allows tumor cells to recover from genotoxic damage induced by chemotherapy or radiation, and activation of alternative signaling pathways can bypass inhibited targets, diminishing the efficacy of molecularly targeted therapies [76].

Therapeutic pressure can also enrich for specialized, resistant subpopulations, including CSCs and dormant (quiescent) cells, which exhibit distinct metabolic programs, elevated DNA repair capacity, and reduced proliferative activity [72,75]. The TME further compounds acquired resistance by providing biochemical, metabolic, and structural support: stromal cells, cancer-associated fibroblasts, extracellular matrix components, hypoxic niches, and immune-modulating factors collectively shield malignant cells from therapeutic pressure and promote adaptive survival responses [77]. Together, these mechanisms contribute to the development of multidrug resistance (MDR), undermine treatment efficacy, and drive tumor recurrence and metastasis.

The heterogeneous nature of cancer, both between patients (inter-tumor heterogeneity) and within individual tumors (intra-tumor heterogeneity), further complicates treatment. Genetically and phenotypically distinct subclonal populations within a tumor may harbor differential drug sensitivities, creating a reservoir of potentially resistant clones primed for selection under therapeutic pressure [23,67,78].

Patients with advanced, metastatic, or relapsed cancers are particularly susceptible to resistance development, especially those with high intra-tumor heterogeneity, such as aggressive solid tumors and many hematologic malignancies [23,79]. Cancers enriched for CSCs, including certain leukemias and rapidly evolving solid tumors, exhibit elevated resistance risk due to enhanced DNA repair, quiescence, and activation of survival pathways [68]. Individuals receiving prolonged or sequential lines of therapy, particularly targeted agents or immunotherapies, face a heightened risk of acquired resistance, as sustained selective pressure promotes expansion of resistant clones through target-site mutations, epigenetic remodeling, and compensatory pathway activation [68,80]. Tumors residing in protective microenvironments, including hypoxic regions and stroma-rich niches, are similarly predisposed to resistance because of metabolic and paracrine support that favors drug evasion [77]. Additional risk factors include host-specific variables that influence drug metabolism and pharmacokinetics, prior exposure to chemoradiation, and cancer types known for rapid phenotypic evolution, such as melanoma, non-small cell lung cancer, and triple-negative breast cancer [62,81].

The clinical trajectory of cancer therapy mirrors that of antimicrobial treatment: early triumphs are often followed by the emergence of resistance and disease relapse. The initial success of early chemotherapeutics, including nitrogen mustard [82] and aminopterin [83], was consistently undermined by rapid resistance development. As in infectious diseases, oncology confronts rapidly proliferating, genetically unstable populations that can adapt to sustained selective pressure. This shared evolutionary dynamic highlights the value of cross-disciplinary insights between antimicrobial resistance and cancer drug resistance research [84].

Understanding the biological foundations of cancer drug resistance is therefore essential for improving patient outcomes and guiding the rational design of next-generation therapies. Strategies such as combination regimens, adaptive dosing, collateral-sensitivity exploitation, and molecular profiling to anticipate resistance trajectories hold promise for addressing this persistent challenge. The following section examines the mechanistic convergences between microbial and cancer drug resistance, identifying shared survival strategies that may inform cross-disciplinary therapeutic development.

## 3. Universal Survival Strategies: Cross-Domain Mechanisms of Drug Resistance

Although antimicrobial and cancer drug resistance arise in distinct biological contexts, both are governed by shared evolutionary and molecular principles that drive adaptation under therapeutic pressure [85]. In both microbial populations and malignant cells, exposure to antimicrobial or anticancer agents selects for subpopulations that can withstand drug-induced stress, enabling their survival and expansion despite treatment. This adaptive process, driven by genetic mutations, epigenetic reprogramming, metabolic rewiring, and activation of compensatory signaling pathways, reflects a high degree of cellular plasticity that allows pathogens and tumors to persist in hostile environments [62].

Across these systems, several recurrent resistance strategies emerge, including enhanced drug efflux, modification or protection of drug targets, enzymatic drug inactivation, epigenetic and transcriptional reprogramming, metabolic adaptation, cellular dormancy, and the formation of protective niches such as bacterial biofilms or extracellular matrix-rich tumor microenvironments. In parallel, phenotypic heterogeneity generates specialized subpopulations, such as bacterial persisters and cancer stem cells, that exhibit intrinsic tolerance to therapy and contribute to relapse or chronic infection [85].

Collectively, these mechanisms highlight drug resistance as a universal biological process rather than a domain-specific phenomenon. Additional processes, including enhanced DNA repair, further support resistance but are typically integrated within broader adaptive strategies rather than functioning as independent mechanisms. The central cross-domain resistance mechanisms discussed in this section are summarized in Table 3.

To avoid overinterpretation of cross-domain similarities, the mechanisms summarized in Table 3 should be interpreted according to the strength of supporting evidence. Efflux-mediated drug export, target modification/mutation, drug inactivation, metabolic adaptation, and microenvironment-mediated protection are supported by substantial mechanistic and clinical evidence in both bacterial infections and cancer. By contrast, epigenetic and regulatory reprogramming, cellular dormancy/persistence, and enhanced DNA repair are strongly supported in one or both domains but often rely more heavily on preclinical, mechanistic, or model-system evidence when considered as directly comparable cross-domain phenomena. Finally, some parallels, such as the conceptual analogy between bacterial persister cells and cancer drug-tolerant persister (DTP) cells, should be regarded as hypothesis-generating frameworks rather than evidence of identical molecular mechanisms. Thus, the comparisons presented in this review emphasize convergent functional strategies while recognizing that the underlying molecular implementation may differ substantially between bacteria and malignant cells. Accordingly, the terms “shared” and “convergent” are used here to indicate functional similarity under therapeutic pressure, not necessarily structural homology or direct mechanistic identity.

**Figure 1:**
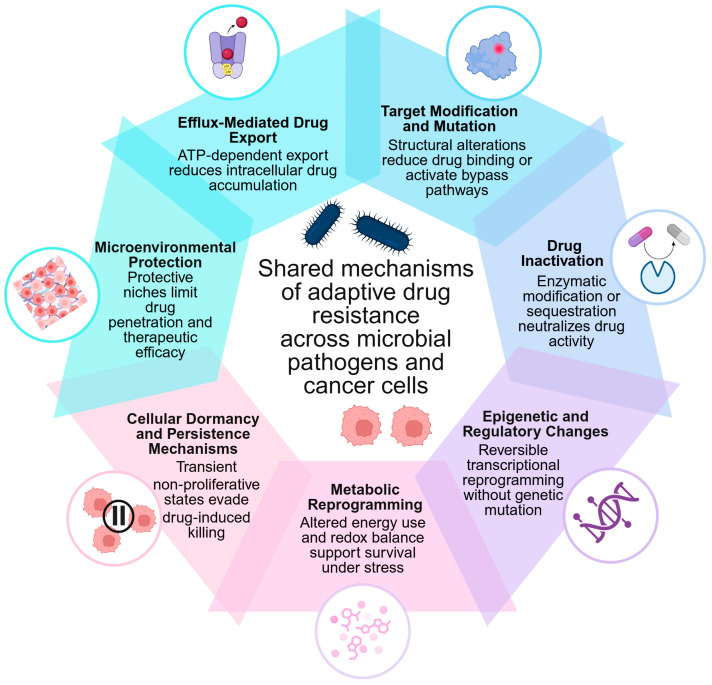
Shared mechanisms of adaptive drug resistance across microbial pathogens and cancer cells. Despite fundamental biological differences, both systems employ convergent strategies to survive therapeutic pressure, including efflux-mediated drug export, target modification and mutation, drug inactivation, epigenetic and regulatory reprogramming, metabolic adaptation, cellular dormancy and persistence, and micro-environmental protection. These mechanisms reflect common evolutionary principles that enable adaptive survival across distinct biological contexts. Created in BioRender. Papaneophytou, C. (2026). https://BioRender.com/5iqphmw (accessed on 8 April 2026).

The following sections analyze each of these cross-domain resistance mechanisms in detail, demonstrating how microbes and tumor cells use functionally analogous strategies to withstand therapeutic pressure (Figure 1 and Table 3). Although both microbes and cancer cells exhibit intrinsic drug resistance, these mechanisms often arise from domain-specific biology, such as bacterial cell envelope structure or tumor microenvironmental features, and are therefore not the main focus of this comparison. Instead, this section focuses primarily on adaptive and acquired mechanisms that represent functionally convergent strategies across both systems.

### 3.1. Efflux-Mediated Drug Export

Efflux-mediated drug export is one of the most conserved and clinically significant mechanisms of drug resistance across both microbial pathogens and cancer [87]. In both systems, the overexpression of membrane transporters actively expels therapeutic agents from the cell, thereby reducing intracellular drug concentrations to subtherapeutic levels and enabling survival under treatment pressure [88].

In bacteria, multidrug efflux pumps from families such as the Major Facilitator Superfamily (MFS), the Resistance-Nodulation-Division (RND) family, and the ABC transporter superfamily mediate resistance to a wide range of antibiotics [89]. Prominent examples include the AcrAB-TolC system in *E. coli* and the MexAB-OprM pump in *P. aeruginosa*, both of which confer resistance to multiple antibiotic classes, including fluoroquinolones, tetracyclines, macrolides, β-lactams, chloramphenicol, and aminoglycosides [90,91,92]. Efflux systems frequently synergize with outer membrane permeability barriers, especially in Gram-negative bacteria, to produce high-level multidrug resistance [93].

In cancer, overexpression of ABC transporter family members is a major contributor to MDR [94,95]. The most clinically relevant efflux transporters include P-gp (encoded by ABCB1/MDR1), BCRP (ABCG2), and the multidrug resistance-associated proteins (MRPs/ABCC family). P-gp is a membrane-associated, ATP-dependent transporter that is physiologically expressed in the gastrointestinal tract, liver, kidney, and at the blood–brain barrier, where it functions to export xenobiotics from cells [96]. In malignancy, ABCB1 is commonly upregulated across multiple cancer types, including ovarian, breast, and lung cancers, as well as acute myeloid leukemia, gliomas, hepatocellular carcinoma, and small cell lung cancer. Overexpression of P-gp reduces intracellular concentrations of chemotherapeutic agents such as paclitaxel, doxorubicin, and vincristine, and is associated with poor clinical outcomes [96,97]. BCRP mediates the efflux of mitoxantrone, topotecan, and SN-38, and its overexpression contributes to resistance in breast cancer, acute lymphoblastic leukemia, and gastrointestinal tumors [98]. MRP1 confers resistance to anthracyclines, vinca alkaloids, methotrexate, and selected tyrosine kinase inhibitors, and is frequently overexpressed in lung cancer, neuroblastoma, and pancreatic cancer [99]. These transporters utilize ATP hydrolysis to expel a broad spectrum of structurally and mechanistically diverse chemotherapeutic agents, including taxanes, anthracyclines, vinca alkaloids, epipodophyllotoxins, tyrosine kinase inhibitors, and PARP inhibitors, thereby limiting drug accumulation and reducing therapeutic efficacy [100,101].

Notably, efflux-mediated resistance can be intrinsic, arising before therapy, or acquired through drug-induced upregulation of transporter expression. A key parallel between microbes and cancer is the broad substrate specificity of efflux pumps, which allows the extrusion of multiple, structurally unrelated drugs. This property makes efflux-mediated resistance particularly challenging, as it can compromise entire therapeutic classes rather than individual agents. Consequently, efflux pump activity is a central driver of treatment failure and multidrug resistance in both infectious diseases and oncology [102]. Therapeutic strategies to overcome efflux-mediated resistance include the development of efflux pump inhibitors (EPIs), structural modification of drugs to evade pump recognition, and advanced drug delivery systems. In the context of infectious diseases, numerous EPIs and membrane permeabilizers have been explored, and structural modifications of antibiotics (e.g., ciprofloxacin derivatives) have shown promise in reducing efflux susceptibility [103,104,105].

However, clinical translation remains limited. In cancer, multiple generations of P-gp inhibitors have been developed, though their use has been hindered by toxicity and pharmacokinetic interactions. More recent advances, including nanoparticle-based carriers, liposomes, polymeric drug conjugates, and biomarker-guided approaches, hold greater promise for overcoming efflux-mediated drug resistance and improving drug delivery to tumor cells [106,107,108]. Although several strategies show potential, the clinical success of EPIs and drug modifications has been more limited in microbiology than in oncology, where advanced delivery systems and molecularly targeted approaches are increasingly integrated into practice [109]. A comprehensive evaluation of drug-specific clinical outcomes lies beyond the scope of this review. Nonetheless, these interventions illustrate a shared therapeutic challenge and highlight efflux inhibition and bypass strategies as necessary cross-disciplinary avenues for overcoming multidrug resistance. Thus, efflux-mediated resistance represents a strong example of functional convergence, but the molecular architecture and regulation of efflux systems differ substantially between bacteria and cancer cells. Bacterial efflux pumps often operate as components of envelope-associated resistance networks, particularly in Gram-negative organisms, whereas cancer-associated ABC transporters function within eukaryotic membrane systems, tissue barriers, and physiological xenobiotic-handling pathways.

### 3.2. Target Modification and Mutation

Modification of drug targets is a key mechanism of resistance shared by both bacterial pathogens and cancer cells. By altering the molecular structures that therapeutic agents bind, cells can reduce drug affinity, interfere with inhibitory interactions, or bypass drug-induced pathway blockade [4]. These alterations arise through genetic mutations, enzymatic modifications, or post-translational changes and serve as powerful adaptive strategies under therapeutic selection [110]. Target modification directly affects the drug’s site of action, rendering some of the most widely used therapeutics ineffective. Organisms that undergo target modifications often exhibit resistance to multiple drugs within the same class, complicating treatment and necessitating alternative or combination therapies [33].

In bacteria, target modification frequently occurs through point mutations in essential genes such as gyrA and parC (fluoroquinolone resistance), rpoB (rifampicin resistance), and genes encoding penicillin-binding proteins (β-lactam resistance) [111,112]. Ribosome-targeting antibiotics, including macrolides and tetracyclines, are often impaired by structural changes in their binding sites; a particularly widespread mechanism is methylation of 23S rRNA mediated by erm methyltransferases, which prevents drug binding to the bacterial ribosome [113,114]. Resistance to glycopeptide and polymyxin antibiotics can arise through chemical modification of cell envelope targets, such as alterations in peptidoglycan precursors or lipid A moieties, respectively [114,115]. These modifications may arise from spontaneous mutations or through the acquisition of resistance genes via HGT, enabling rapid dissemination across bacterial populations. Target modification can also occur through target replacement or bypass, in which bacteria acquire or upregulate alternative enzymes that perform the same essential function but are insensitive to the drug (reviewed in [48]).

Cancer cells exhibit analogous mechanisms of resistance through somatic mutations or structural alterations in therapeutic targets. Classic examples include the BCR-ABL T315I mutation conferring imatinib resistance, EGFR mutations (e.g., T790M, C797S) driving tyrosine kinase inhibitor resistance, and activating or bypass mutations in KRAS or BRAF that negate the effect of upstream targeted therapies [69,116]. Cancer cells may also express alternative splice variants (such as truncated androgen receptor isoforms) or increase target abundance, thereby diminishing inhibitor potency. Both microbes and cancer cells can also modify drug targets through post-translational modifications. In bacteria, methylation of ribosomal RNA prevents antibiotic binding, and other enzymatic changes modulate cell envelope targets [114]. In cancer, phosphorylation, acetylation, or conformational changes in kinases and receptors can compromise drug-target interactions and promote resistance [117]. A notable convergence is the emergence of on-target and off-target resistance. On-target resistance involves direct changes to the drug-binding site, such as mutations in the kinase domain or alterations in penicillin-binding proteins. Off-target resistance reflects compensatory activation of alternative pathways that bypass the inhibited target, a phenomenon observed in both bacterial regulatory networks and cancer signaling cascades [80,118,119]. Together, these mechanisms highlight the shared evolutionary pressure imposed by therapeutic agents and underscore the need for ongoing molecular surveillance in both infectious diseases and oncology, as well as the development of next-generation inhibitors that target mutated or structurally altered proteins [120]. Thus, target modification represents a clear shared resistance principle, but its biological implementation differs across domains. In bacteria, target alteration often arises through point mutations, enzymatic modification, or horizontally acquired resistance determinants, whereas in cancer it typically reflects somatic evolution, clonal selection, altered signaling architecture, and pathway redundancy within a single host.

### 3.3. Drug Inactivation

Drug inactivation is a major mechanism of resistance that undermines therapeutic efficacy in both microbial pathogens and cancer. Although the underlying biochemical strategies differ between these biological systems, the functional outcome is the same: therapeutic agents are neutralized before reaching or engaging their intended targets.

In bacteria, enzymatic inactivation is one of the most widespread and clinically impactful mechanisms of AMR, operating through two principal strategies. The first is drug degradation via hydrolysis: the most prominent example is β-lactamases, a large family of enzymes that hydrolyze the β-lactam ring of penicillins, cephalosporins, and carbapenems. Extended-spectrum β-lactamases (ESBLs) and carbapenemases (e.g., KPC, NDM) confer high-level resistance across multiple β-lactam classes [121]. Additional hydrolytic mechanisms include the degradation of tetracyclines via *tetX*-encoded monooxygenases [122]. The second strategy is drug modification through chemical group transfer: bacteria frequently employ acetyltransferases, phosphotransferases, and adenyltransferases that modify aminoglycosides, chloramphenicol, streptogramins, and certain fluoroquinolones, preventing drug binding to their targets [123,124]. These resistance determinants are often mobilized via HGT, including plasmids, integrons, and transposons, facilitating rapid dissemination within and between species [125]. Recent work by Li et al. [126] further demonstrated that drug inactivation is a major determinant of antibiotic inhibition phenotypes. Using growth curve modeling in *E. coli*, the authors showed that inactivation consistently produced characteristic lag-phase phenotypes distinct from efflux-mediated resistance, suggesting that growth kinetics can serve as a rapid functional predictor of drug-inactivating bacteria.

In cancer, drug inactivation primarily involves the upregulation of detoxifying pathways that neutralize or metabolize chemotherapeutic agents. The deactivation of many chemotherapeutic agents is regulated by drug-metabolizing enzymes (DMEs) [127]. Dysregulation of DMEs and metabolic signaling pathways, leading to enhanced detoxification of active drugs or failure in the conversion of prodrugs into active metabolites, represents a major mechanism of chemoresistance [128]. Metabolism-associated drug inactivation represents a central axis of chemoresistance, arising either from impaired prodrug activation or accelerated detoxification of active agents [129]. Several frontline chemotherapeutics depend on metabolic activation, and loss of the activating enzymes directly diminishes cytotoxic efficacy. For example, irinotecan requires conversion to SN-38 by carboxylesterases [130], 5-fluorouracil (5-FU) is activated by thymidine phosphorylase [131], and cytarabine (Ara-C) must be phosphorylated by deoxycytidine kinase (DCK) to generate Ara-CTP, with DCK deficiency or mutation repeatedly linked to Ara-C resistance and cross-resistance to other nucleoside analogs such as gemcitabine [132]. In parallel, multiple enzyme families catalyze drug detoxification, thereby lowering intracellular drug exposure. Aldehyde dehydrogenase (ALDH) isoforms, particularly ALDH1A1 and ALDH3A1, inactivate nitrogen mustards, including cyclophosphamide and ifosfamide, and ALDH activity is also associated with cancer stem cell survival and chemoresistance [127]. Cytochrome P450 (CYP450) enzymes, which mediate the phase I oxidation of many clinically used drugs, have been implicated in resistance to several anticancer agents. In particular, CYP1B1, CYP2C8, CYP3A4, and CYP3A5 have been associated with reduced sensitivity to taxanes, mitoxantrone, flutamide, and gemcitabine, although the extent to which CYP1B1 directly contributes to drug inactivation remains uncertain [133,134]. Conjugating enzymes involved in phase II metabolism further contribute to drug resistance by enhancing detoxification and promoting drug elimination. Glutathione S-transferases (GSTs), including GSTA1, GSTM2, and GSTP1, promote glutathione conjugation of platinum agents, doxorubicin, and alkylators, with GSTP1 overexpression strongly linked to platinum-GSH conjugate formation and reduced drug efficacy [135,136]. Notably, after GSH conjugation, the conjugated drugs may become substrates for ABC transporters, especially the MDR-associated proteins, and are actively expelled from cancer cells [137].

Similarly, UDP-glucuronosyltransferases (UGTs), which are involved in phase II metabolism, contribute to drug resistance by inactivating therapeutic agents through glucuronidation. UGT1A1 is especially significant because it glucuronidates and inactivates SN-38, the active metabolite of irinotecan, thus decreasing treatment effectiveness [138]. UGTs have also been linked to resistance to HSP90 inhibitors, erlotinib, and even monoclonal antibodies such as nivolumab, either by directly metabolizing the drugs or by indirectly affecting signaling pathways [139,140,141]. Collectively, dysregulation of these drug-metabolizing enzymes reduces active drug levels, drives multidrug resistance, and highlights metabolic enzymes as both predictive biomarkers and therapeutic targets in overcoming chemoresistance.

Critically, drug inactivation in cancer is not restricted to tumor cells themselves. Emerging evidence demonstrates that the tumor-associated microbiota can metabolize chemotherapeutics into inactive metabolites; for example, intratumoral *Gammaproteobacteria* in pancreatic cancer can enzymatically inactivate gemcitabine, contributing to treatment failure [142]. This microbiome-mediated drug inactivation introduces a community-level dimension to cancer drug resistance that parallels the horizontal dissemination of resistance genes in bacterial populations [143]. Although the underlying enzymatic systems differ, the functional consequence is similar: reduced availability of the active drug at its intended site of action. In bacteria, drug inactivation commonly depends on specialized resistance enzymes, such as β-lactamases or aminoglycoside-modifying enzymes, that directly degrade or chemically modify antibiotics. In cancer, by contrast, resistance more often reflects the upregulation of endogenous metabolic and detoxification pathways, including GSTs, ALDHs, CYP450 enzymes, and UGTs, rather than the evolution of drug-specific inactivating enzymes [144]. The tumor microbiome adds a further layer of complexity by enabling community-mediated drug metabolism within the malignant niche.

Despite these biological differences, microbes and tumor cells converge on several functional principles of drug inactivation: enzymatic modification or degradation of therapeutic agents, reduced active drug concentration at the target site, selection for enzyme upregulation, and class-wide resistance due to broad enzyme substrate specificity. Approaches to counter drug inactivation span both domains and include enzyme inhibitors such as β-lactamase inhibitors (clavulanate, tazobactam, avibactam) that restore antibiotic activity; metabolic pathway targeting in cancer through inhibition of GSTs, ALDHs, or CYP-mediated metabolism to enhance drug exposure; nanoparticle and liposomal delivery systems that protect drugs from enzymatic modification; structural modification of drugs to evade enzymatic recognition; and microbiome-targeted therapies, including antibiotics or bacteriophages, to eliminate drug-inactivating intratumoral bacteria [139,145]. Accordingly, drug inactivation should be viewed as a functionally convergent strategy rather than a mechanistically identical process: bacteria rely largely on specialized resistance enzymes, whereas cancer cells primarily exploit endogenous metabolic and detoxification networks.

### 3.4. Epigenetic and Regulatory Changes

Epigenetic and regulatory mechanisms form a powerful layer of adaptive resistance shared by both bacterial pathogens and cancer cells. Unlike mutations, epigenetic changes modify gene expression without altering the DNA sequence, enabling rapid, reversible, and heritable phenotypic shifts and plasticity. This versatility allows cells to enter drug-tolerant states, regulate stress responses, and reprogram survival pathways under therapeutic pressure [119,146]. In cancer, epigenetic mechanisms are key drivers of drug resistance. These include DNA methylation, histone modifications, chromatin remodeling, nucleosome repositioning, and non-coding RNA regulation [147]. Aberrant DNA methylation mediated by DNMT1, DNMT3A, and DNMT3B can silence tumor suppressor genes, upregulate drug efflux pumps, or activate survival pathways [148,149,150]. Histone-modifying enzymes, including histone deacetylases (HDACs), histone acetyltransferases (HATs), methyltransferases, demethylases (e.g., KDM family members), and chromatin remodelers, drive transcriptomic reprogramming that supports drug tolerance and cancer progression [151,152]. Epigenetic plasticity enables tumor cells to enter DTP states: slow-cycling, therapy-refractory subpopulations that survive targeted therapy and can later re-expand or acquire permanent genetic resistance [153,154]. These transient states, mediated by chromatin remodeling and non-coding RNAs, contribute to minimal residual disease and tumor recurrence after therapy withdrawal. Because epigenetic states are reversible, targeting epigenetic regulators (e.g., DNMT inhibitors, HDAC inhibitors, BET inhibitors, histone demethylase inhibitors, RNA-targeted drugs) is a promising strategy to resensitize tumors and prevent the evolution of permanent resistance [155,156]. Although historically underappreciated, bacteria also utilize epigenetic systems to regulate antibiotic susceptibility and adaptive resistance.

Bacterial epigenetic mechanisms primarily include DNA methylation, phase variation, non-coding RNAs, and global transcriptional regulators that dynamically modify gene expression during antibiotic exposure [157,158]. Cytosine or adenine methylation by DNA methyltransferases (Mtases) can modulate RNA polymerase binding, activate or repress promoters, and alter transcription of genes involved in efflux, cell envelope remodeling, virulence, and stress responses [157]. Differential methylation patterns have been observed in drug-resistant *Mycobacterium tuberculosis* and other pathogens, implicating epigenetic inheritance in antimicrobial tolerance [159,160]. Phase variation allows bacteria to switch gene expression on or off through reversible methylation-dependent promoter control, generating rapid population heterogeneity that can alter LPS O-antigen expression in *Salmonella enterica*, pilus and adhesin expression in *E. coli* (pap operon), and cell-surface structures in *Neisseria meningitidis*, *Haemophilus influenzae*, and *Helicobacter pylori* [161,162,163]. Phase-variable Mtases can also influence susceptibility to certain antibiotics by shifting expression patterns of efflux pumps, porins, and metabolic pathways. Epigenetic mechanisms further contribute to heteroresistance and the emergence of persister cells, which are non-growing, highly tolerant bacterial subpopulations that survive antibiotic exposure and repopulate after drug removal. This reversible, non-genetic tolerance cannot be fully explained by mutations and increasingly appears to involve methylation-dependent transcriptional programs [164,165,166]. Small RNAs and global regulators such as MarA, SoxS, and RamA orchestrate antibiotic tolerance by modulating efflux pump expression, oxidative stress responses, and membrane remodeling [155].

Despite differences in molecular machinery, microbes and cancer cells share several epigenetic resistance principles: rapid, reversible changes in gene expression; induction of DTP populations; modulation of efflux pumps, metabolism, and survival pathways; phenotypic heterogeneity under drug stress; and heritable yet non-mutational resistance states. However, key differences remain. In bacteria, epigenetics mainly regulates environmentally responsive gene circuits, phase variation, and community behaviors such as biofilm formation [167]. In cancer, epigenetics drives complex chromatin reprogramming, lineage transitions, epithelial–mesenchymal transition (EMT), and the creation of stable drug-tolerant states [154]. Bacteria utilize Mtases as their main epigenetic effectors [168], while cancer depends heavily on chromatin-modifying enzymes [169].

Because epigenetic changes are reversible in both systems, targeting these mecha-nisms offers a promising therapeutic approach across fields: epigenetic drugs, including DNMT inhibitors, HDAC inhibitors, and BET inhibitors, can reverse drug tolerance in cancer [170]; targeting regulatory pathways such as MarA/SoxS/RamA may influence bacterial tolerance [171]; inhibiting phase variation or Mtase activity may limit bacterial adaptive plasticity [172]; disrupting epigenetic plasticity may resensitize persister populations [173]; and combining epigenetic modulation with antimicrobial or anticancer treatments may delay the emergence of resistant clones. Thus, the parallel between microbes and cancer lies primarily in reversible regulatory plasticity and phenotypic adaptation, rather than in shared epigenetic machinery. This distinction reinforces the view that epigenetic and regulatory resistance represents a functionally convergent strategy implemented through fundamentally different molecular systems.

### 3.5. Metabolic Reprogramming and Detoxification Pathways

Metabolic reprogramming is a key adaptive strategy enabling both microbial pathogens and cancer cells to modify their biochemical processes to withstand therapeutic pressure [174,175]. Beyond the direct inactivation of drugs discussed above (Section 3.3), broader shifts in central carbon metabolism, energy production, redox balance, and biosynthesis can greatly influence drug uptake, activation, and efficacy. In both areas, metabolic flexibility enables cells to develop resistant or tolerant phenotypes that traditional susceptibility tests might not easily detect.

In bacteria, the metabolic state is a key factor influencing antibiotic susceptibility. Most bactericidal antibiotics, including β-lactams, aminoglycosides, and fluoroquinolones, are most effective against actively growing, metabolically active cells. Bacteria that switch to lower metabolic states due to nutrient scarcity, enter the stationary phase, or activate the stringent response mediated by the alarmone (p)ppGpp, exhibit greatly increased tolerance to these drugs [85,176]. Aminoglycosides, for example, require an active proton motive force (PMF) for cellular uptake; bacteria that downregulate aerobic respiration or shift to fermentative metabolism effectively reduce aminoglycoside accumulation and killing [177]. Small colony variants (SCVs), frequently associated with chronic and relapsing infections caused by *S. aureus* and *P. aeruginosa*, exemplify metabolic reprogramming as a resistance mechanism. SCVs harbor defects in electron transport chain components (e.g., menadione or hemin biosynthesis), resulting in slow growth, reduced membrane potential, and diminished susceptibility to aminoglycosides and other antibiotics that depend on active cellular metabolism for uptake or activity [178]. Additionally, bacteria can increase their antioxidant defenses, including catalase, superoxide dismutase, and alkyl hydroperoxide reductase, to reduce oxidative stress induced by bactericidal antibiotics, thereby enhancing their survival under treatment pressure [179].

In cancer, metabolic reprogramming is recognized as a hallmark of malignancy and a key factor in drug resistance [180]. The Warburg effect, which refers to the preference for glycolysis over oxidative phosphorylation (OXPHOS) even in the presence of sufficient oxygen, supplies tumor cells with metabolic intermediates for biosynthesis and produces NADPH via the pentose phosphate pathway to maintain redox balance [181,182]. Resistant tumor cells often display increased metabolic flexibility, switching between glycolysis and OXPHOS in response to drug exposure and microenvironmental changes [183]. Fatty acid oxidation (FAO) is an especially important resistance-related metabolic pathway, providing an alternative energy source for drug-resistant cancer cells and CSCs [184]. Glutamine addiction, driven by heightened demand for nitrogen and carbon intermediates, supports nucleotide synthesis, amino acid production, and glutathione synthesis, linking metabolic reprogramming to biosynthetic capacity and antioxidant defense. This enhances the glutathione system and other redox-buffering pathways, helping tumor cells to withstand oxidative damage from chemotherapy and radiation [185], complementing the detoxification enzymes discussed above. Hypoxia-inducible factor 1α (HIF-1α) acts as a master regulator of metabolic adaptation in the TME, promoting glycolytic gene expression, downregulating mitochondrial respiration, and supporting survival under hypoxic conditions that are typical of many solid tumors [186].

A key intersection between microbial and cancer drug resistance is the use of metabolic flexibility as a survival strategy. In both systems, decreasing metabolic activity or shifting toward alternative energy pathways can reduce drug uptake, limit drug activation, and increase tolerance. The upregulation of antioxidant and detoxification systems, whether through catalase and superoxide dismutase in bacteria or the glutathione system and NADPH-producing pathways in cancer, helps protect cells from drug-induced oxidative damage. The ability to switch between metabolic states, such as actively growing versus dormant or metabolically suppressed phenotypes, is a common way for both pathogens and tumor cells to evade therapies that preferentially target metabolically active cells. However, the biological context of metabolic adaptation differs substantially between the two domains. In bacteria, metabolic shifts often reflect rapid responses to nutrient limitation, respiration state, stress signaling, and antibiotic uptake requirements, whereas in cancer, metabolic reprogramming is embedded within oncogenic signaling, tumor microenvironmental constraints, and systemic host metabolism. Thus, metabolic reprogramming represents a functionally convergent resistance strategy, but the regulatory networks and physiological context governing this adaptation differ markedly between bacterial pathogens and malignant cells. These findings suggest that targeting metabolic dependencies could enhance the efficacy of traditional antimicrobial and anticancer treatments.

### 3.6. Cellular Dormancy and Persistence Mechanisms

Cellular dormancy and persistence represent among the most clinically consequential mechanisms of drug resistance across both bacterial infections [187] and cancer [188]. In both systems, a subset of cells can enter a quiescent or slow-cycling state characterized by dramatically reduced metabolic activity and proliferation, rendering them inherently tolerant to therapies that target actively dividing populations. These dormant subpopulations survive treatment and can subsequently reactivate, driving disease relapse, chronic infection, or cancer recurrence long after an apparently successful therapeutic response.

In bacteria, persister cells are phenotypic variants within a genetically susceptible population that survive lethal antibiotic concentrations without harboring resistance mutations [189]. First described by Bigger in 1944 [190] in the context of penicillin treatment for staphylococcal infections, persisters are a subpopulation that, either stochastically or due to stress, enter a dormant, non-replicating state [191]. Multiple molecular mechanisms contribute to persister formation, including toxin-antitoxin (TA) modules such as HipA/HipB and MazEF, the stringent response mediated by (p)ppGpp signaling, and the SOS DNA damage response [192]. These pathways work together to inhibit macromolecular synthesis and metabolic activity, rendering cells phenotypically tolerant to bactericidal agents. Besides classical persisters, bacteria can also enter viable but non-culturable (VBNC) states, where cells remain metabolically active at low levels but do not grow on standard culture media, complicating clinical detection and monitoring of treatment [193]. SCVs, discussed above in relation to metabolic reprogramming, are another form of phenotypic dormancy associated with chronic and relapsing infections [194]. The clinical significance of bacterial persistence is considerable: persister-induced tolerance is involved in the relapse of tuberculosis, recurrent urinary tract infections, chronic lung infections in cystic fibrosis, and treatment-refractory endocarditis [191].

Cancer cells employ analogous dormancy strategies to survive therapeutic exposure. DTP cells, first characterized in the context of EGFR-targeted therapy in non-small cell lung cancer, represent a small, reversibly quiescent subpopulation that survives initial drug exposure through non-genetic mechanisms, including chromatin remodeling, altered lipid metabolism, and upregulation of antioxidant pathways [153]. DTPs can remain in a slow-cycling state for extended periods and subsequently re-enter the cell cycle, often acquiring additional genetic or epigenetic changes that confer permanent resistance [195]. CSCs represent a related but distinct population characterized by self-renewal capacity, enhanced DNA repair, elevated expression of ABC efflux transporters, and activation of developmental signaling pathways, including Wnt, Notch, and Hedgehog. CSCs are enriched in minimal residual disease following chemotherapy and are thought to serve as a reservoir for tumor recurrence and metastatic seeding [68]. Tumor dormancy at the organismal level, in which disseminated tumor cells persist in a quiescent state in distant organs for months to years before reactivating as overt metastases, represents another clinically significant manifestation of cellular dormancy. Mechanisms underlying tumor dormancy include immune surveillance, angiogenic insufficiency, and cell-intrinsic quiescence programs mediated by p38/MAPK signaling, DEC2, and NR2F1 [196].

The parallels between bacterial persistence and cancer cell dormancy are striking. In both contexts, a phenotypically distinct subpopulation enters a quiescent, drug-tolerant state that is not explained by genetic resistance mechanisms. These dormant cells survive treatment, persist as residual disease, and serve as a reservoir for relapse upon cessation or modification of therapy. The molecular regulators differ in specificity (TA modules and (p)ppGpp in bacteria; chromatin remodelers and developmental signaling in cancer), yet the functional logic is conserved: suppression of growth-related processes to create a protected state that outlasts drug exposure. Phenotypic heterogeneity within both bacterial and tumor populations ensures that dormant cells are present even before treatment begins, providing an immediate survival advantage under sudden therapeutic selection [62,85].

### 3.7. Microenvironmental Influences on Drug Efficacy

The microenvironment where pathogens or tumor cells live strongly influences how well drugs work, often providing physical, biochemical, and immune defenses that can lead to treatment failure. In both infections and cancer, cells do not exist alone but are part of organized communities or tissue niches that influence drug access, cell metabolism, immune system recognition, and stress responses. These microenvironmental factors are a crucial and often overlooked part of drug resistance [197,198].

In bacteria, the biofilm represents the archetypal protective microenvironment. Biofilms are structured communities of bacteria encased in a self-produced extracellular polymeric substance (EPS) matrix composed of polysaccharides, proteins, extracellular DNA, and lipids [199]. The EPS matrix restricts antibiotic diffusion, creates concentration gradients that expose interior cells to subinhibitory drug levels, and generates metabolic heterogeneity, with actively growing cells at the biofilm periphery and metabolically dormant, persister-enriched cells in deeper layers. Biofilm-associated bacteria can exhibit antibiotic tolerance levels 100- to 1000-fold higher than their planktonic counterparts, making biofilm infections notoriously difficult to eradicate [200]. Clinically, biofilms are implicated in a wide range of chronic infections, including prosthetic joint infections, catheter-associated urinary tract infections, ventilator-associated pneumonia, chronic wound infections, and endocarditis. Beyond biofilms, intracellular survival represents another microenvironmental strategy employed by pathogens such as *Mycobacterium tuberculosis*, *Salmonella* spp., and *S. aureus*, which can persist within macrophages or epithelial cells. The intracellular niche provides protection from extracellular antibiotics, immune effectors, and environmental stresses, while simultaneously modulating bacterial gene expression and metabolic programs to promote long-term survival [201,202]. Additionally, local tissue conditions at the infection site, including low pH, reduced oxygen tension, high osmolality, and the presence of host defense molecules, can alter antibiotic activity and bacterial physiology, thereby promoting tolerance [203].

In cancer, the TME plays an equally central role in drug resistance. The TME is a complex ecosystem comprising tumor cells, cancer-associated fibroblasts (CAFs), tumor-associated macrophages (TAMs), endothelial cells, pericytes, immune cells, extracellular matrix (ECM) components, and soluble signaling molecules [204,205]. Each of these elements can contribute to resistance through distinct mechanisms. The ECM acts as a physical barrier that impedes drug penetration into the tumor interior, while CAFs secrete growth factors, cytokines, and metabolites that promote tumor cell survival and activate resistance-associated signaling pathways [206]. Hypoxia, a hallmark of solid tumors caused by abnormal blood vessels and rapid cell growth, triggers HIF-1α-dependent transcription programs that support metabolic changes, blood vessel formation, and resistance to both chemotherapy and radiation. The acidic environment outside the cells, resulting from Warburg glycolysis, can lower the uptake of weakly basic drugs through ion trapping, further reducing treatment effectiveness [207,208].

Immune components of the TME, including regulatory T cells (Tregs), myeloid-derived suppressor cells (MDSCs), and TAMs with immunosuppressive phenotypes, create an immune-privileged niche that undermines the efficacy of immunotherapies and reduces immune-mediated tumor clearance. Paracrine signaling between stromal and tumor cells, mediated by factors such as HGF, IL-6, and TGF-β, can activate bypass signaling pathways that sustain tumor cell viability despite targeted inhibitors [209].

The microenvironmental strategies employed by bacteria and cancer cells converge on several shared principles. In both systems, physical barriers, such as the EPS matrix in biofilms and the ECM in tumors, restrict drug penetration and create concentration gradients that expose cells to sublethal drug levels, promoting the selection of tolerant or resistant subpopulations. Metabolic gradients within both biofilms and solid tumors generate heterogeneous microenvironments that harbor dormant, drug-tolerant cells in nutrient-poor and hypoxic regions. Community-level signaling, including bacterial quorum sensing and tumor paracrine signaling, coordinates collective responses to therapeutic stress. Immune evasion is also a shared feature: biofilms resist phagocytosis and complement-mediated killing, while the TME suppresses antitumor immunity through multiple cellular and molecular mechanisms. Both biofilms and the TME can serve as reservoirs for residual disease, sustaining small populations of surviving cells that may repopulate after treatment cessation [85,210,211,212]. However, these protective niches differ fundamentally in composition, organization, and host interaction. Bacterial biofilms are microbial community structures embedded in a self-produced extracellular polymeric matrix, whereas the TME is a complex host-derived tissue ecosystem involving stromal, vascular, immune, and extracellular matrix components. Thus, biofilms and tumor microenvironments represent functionally convergent protective niches rather than mechanistically identical systems. These parallels suggest that strategies to disrupt protective microenvironments, whether through biofilm-dispersing agents, ECM-degrading enzymes, vascular normalization, or immunomodulatory interventions, may enhance drug delivery and restore therapeutic efficacy across both domains. Such approaches are discussed in greater detail in Section 4.

## 4. Translational Insights: Cross-Disciplinary Therapeutic Strategies Against Drug Resistance

The striking mechanistic similarities between microbial and cancer drug resistance provide a strong foundation for cross-disciplinary therapeutic innovation. Despite fundamental differences in biological organization, both systems are governed by shared evolutionary principles that drive adaptation under pharmacological pressure. Microbial pathogens and tumor cells employ convergent strategies, including efflux-mediated drug export, target modification, metabolic reprogramming, epigenetic plasticity, and protective microenvironments, to evade treatment. These parallels position drug resistance as a universal biological process rather than a domain-specific phenomenon, opening opportunities for unified intervention strategies. Accordingly, insights from microbial resistance, particularly in bacteria, can inform cancer therapy, while advances in oncology may guide antimicrobial innovation.

From a translational perspective, however, the strategies discussed below differ substantially in clinical maturity. Combination therapy is the most clinically established approach in both infectious diseases and oncology, with clear efficacy in tuberculosis, HIV, HCV, selected bacterial infections, and multiple cancer settings. β-lactam/β-lactamase inhibitor combinations are clinically validated in antimicrobial therapy, while several nanomedicine formulations and immune checkpoint inhibitors are established components of cancer treatment [12,213]. By contrast, approaches such as efflux pump inhibition have strong mechanistic support but have faced major translational barriers, including toxicity, pharmacokinetic interactions, transporter redundancy, and insufficient biomarker-guided patient selection [88,214]. Other strategies, including quorum-sensing (QS) inhibition [215], anti-persister “wake-and-kill” approaches [216], broad anti-biofilm interventions [217], microbiome-targeted drug-inactivation strategies [218], and many repurposed agents, remain largely experimental or preclinical. Across both fields, successful translation will require rigorous validation in clinically relevant models, pharmacokinetic–pharmacodynamic optimization, resistance biomarkers for patient or pathogen stratification, and carefully designed combination or sequencing strategies.

**Figure 2:**
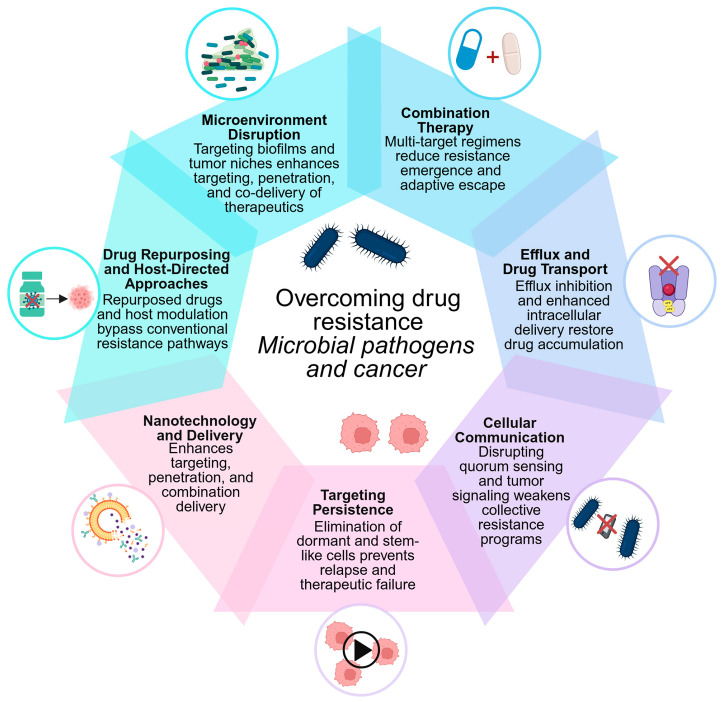
Cross-disciplinary therapeutic strategies to overcome drug resistance across microbial infections and cancer. These approaches target shared adaptive survival mechanisms through combination therapy, efflux and transport inhibition, disruption of cellular communication, elimination of persistent cell populations, nanotechnology-enabled delivery systems, drug repurposing, host-directed interventions, and microenvironmental modulation. Together, these strategies illustrate how mechanistic insights from microbiology and oncology can be translated into integrated therapeutic frameworks to improve treatment durability across both domains. Created in BioRender. Papaneophytou, C. (2026) https://BioRender.com/stenref (accessed on 8 April 2026).

Representative examples of these approaches are summarized in Table 4, and their conceptual framework is illustrated in Figure 2. The following subsections examine key translational strategies to prevent, delay, or overcome drug resistance across both domains.

### 4.1. Combination Therapy as a Resistance-Management Strategy

Combination therapy is the most established and widely validated strategy for managing drug resistance in both infectious diseases and cancer. The core idea is that targeting multiple biological pathways or molecular targets simultaneously decreases the chance that any resistant subgroup can survive treatment, since different resistance mechanisms would need to occur within the same cell or population [213,224].

In infectious diseases, the rationale for combination therapy was first demonstrated in tuberculosis, where early multi-drug regimens, beginning with streptomycin and para-aminosalicylic acid in 1950, dramatically reduced the emergence of resistance compared with monotherapy [225]. This foundational insight later led to the development of modern multi-drug regimens, such as the combination of isoniazid, rifampicin, pyrazinamide, and ethambutol, which remain the standard of care and continue to suppress resistance far more effectively than single-agent therapy [225]. This approach was subsequently adopted for HIV-1 infection, where combination antiretroviral therapy (cART) transformed a fatal disease into a manageable chronic condition, and for HCV infection, where multi-agent DAA regimens now achieve cure rates exceeding 95% [226]. In bacterial infections, combination strategies are employed for empiric treatment of severe sepsis, endocarditis, and infections caused by MDR organisms, in which agents with complementary mechanisms (e.g., β-lactam plus aminoglycoside or β-lactam plus β-lactamase inhibitor) are combined to broaden coverage and suppress resistance selection [227].

In oncology, combination regimens have been a key therapeutic approach since the development of MOPP (mechlorethamine, vincristine, procarbazine, prednisone) for Hodgkin lymphoma in the 1960s [228]. Modern combination strategies go far beyond cytotoxic chemotherapy to include targeted agents with immunotherapies, dual pathway inhibition to prevent bypass resistance, and chemo-immunotherapy combinations. Examples include the combination of BRAF and MEK inhibitors in melanoma (addressing the rapid resistance observed with single-agent BRAF inhibition), concurrent immune checkpoint blockade with anti-PD-1 and anti-CTLA-4 antibodies, and the pairing of targeted agents with chemotherapy in breast and lung cancers [229]. The design of effective combination regimens increasingly relies on molecular profiling and predictive biomarkers to identify patients most likely to benefit and to anticipate resistance trajectories [230]. A critical translational insight from infectious disease is the concept of mutant prevention concentration (MPC), the drug concentration above which the selection of first-step resistant mutants is minimized [231]. This PK-PD framework, long established in antimicrobial therapy, is increasingly being translated into oncology. Adaptive therapy applies analogous principles by modulating drug exposure to suppress resistant clones while preserving drug-sensitive competitors, thereby creating a therapeutic window that minimizes resistance evolution. As such, it represents a bidirectional innovation that draws on ecological principles originally developed in microbial population dynamics and is now being adapted to oncology [232]. Together, these approaches illustrate how cross-disciplinary exchange between infectious disease and oncology can advance the rational design of combination regimens that delay or prevent the evolution of resistance.

### 4.2. Targeting Efflux and Drug Transport Mechanisms

The therapeutic targeting of efflux-mediated resistance, whose mechanistic basis was discussed in Section 3.1, represents a long-pursued yet incompletely realized translational goal in both antimicrobial therapy and oncology. While the molecular understanding of efflux pumps is now well advanced, converting this knowledge into clinically effective interventions has proven challenging in both domains, offering instructive lessons for cross-disciplinary development [104].

In antimicrobial therapy, EPIs have been extensively investigated as adjuvants to restore antibiotic efficacy [104]. Compounds such as phenylalanine-arginine β-naphthylamide (PAβN) and 1-(1-naphthylmethyl)-piperazine (NMP) have demonstrated in vitro activity against RND-family pumps in Gram-negative bacteria, and natural product-derived inhibitors (e.g., reserpine, berberine) have shown activity against Gram-positive efflux systems [233]. However, clinical translation has been limited by toxicity, poor pharmacokinetics, and substrate redundancy in bacterial efflux systems, which often express multiple pump families with overlapping specificities [103,104]. More recent approaches include the development of antibiotic-EPI hybrid molecules, in which an efflux inhibitor is covalently linked to an antibiotic scaffold, and the use of membrane permeabilizers that enhance intracellular drug accumulation by disrupting outer membrane integrity in Gram-negative bacteria [234].

In oncology, three generations of P-gp (ABCB1) inhibitors have been developed. First-generation agents (verapamil, cyclosporin A) lacked specificity and caused significant toxicity. Second-generation inhibitors (valspodar, biricodar) improved selectivity but also interfered with drug metabolism by inhibiting CYP3A4, complicating pharmacokinetics. Third-generation agents (tariquidar, zosuquidar, elacridar) demonstrated high potency and selectivity for ABC transporters; however, clinical trials have yielded disappointing results, largely due to patient selection challenges, compensatory upregulation of alternative transporters, and the difficulty of achieving sustained in vivo efflux inhibition [100,235]. Emerging strategies in oncology now focus on nanoparticle-based co-delivery of chemotherapeutics and efflux inhibitors, antibody-drug conjugates that bypass transporter-mediated efflux through receptor-mediated endocytosis, and the use of transcriptional or epigenetic inhibitors to suppress efflux pump expression at its source [107,236].

The parallel challenges encountered in both fields suggest several shared lessons. First, single-target efflux inhibition is unlikely to succeed when multiple efflux systems with overlapping substrate profiles are co-expressed. Second, combination approaches that pair efflux inhibition with complementary strategies (membrane disruption, transcriptional suppression, nanoparticle delivery) are more likely to achieve durable efficacy. Third, biomarker-guided identification of patients or infections with efflux-dominated resistance phenotypes may improve clinical trial design and therapeutic outcomes in both domains [88].

### 4.3. Disrupting Cellular Communication: Quorum Sensing and Tumor Signaling

Intercellular communication is essential for coordinating collective resistance responses in both microbial and tumor populations. Disrupting these communication networks represents an attractive therapeutic strategy, as it targets the cooperative behaviors that promote resistance rather than directly killing individual cells, potentially reducing the selective pressure that drives resistance evolution [237,238].

In bacteria, quorum sensing (QS) is a cell-density-dependent signaling system in which small diffusible molecules (autoinducers) coordinate population-wide gene expression programs, including biofilm formation, virulence factor production, and efflux pump expression [238]. The major QS systems include acyl-homoserine lactone (AHL)-based signaling in Gram-negative bacteria (e.g., the las and rhl systems in *P. aeruginosa*), autoinducer-2 (AI-2) interspecies signaling, and autoinducing peptide (AIP) systems in Gram-positive organisms [239]. QS inhibitors (QSIs) aim to disrupt these signaling networks without directly killing bacteria, thereby reducing virulence and biofilm-mediated resistance while, in theory, imposing lower selective pressure for resistance evolution [240]. Experimental QSIs include AHL analogs, lactonases that degrade autoinducers, and small molecules targeting QS receptors such as LasR and RhlR [241]. Recent work from our group and others has demonstrated that phytochemicals represent a particularly rich and underexploited source of QSIs. Plant-derived extracts, including those from *Origanum vulgare*, *Rosmarinus officinalis*, and *Salvia officinalis*, have been shown to suppress AI-2 signaling, inhibit biofilm formation, and impair motility in Gram-negative bacteria without affecting growth, consistent with true quorum-quenching activity [242]. Complementary studies evaluating phytochemicals as LuxS/AI-2 pathway inhibitors further highlight their capacity to disrupt intercellular communication networks central to virulence and collective resistance behaviors [243]. Preclinical studies have demonstrated that QS inhibition can resensitize biofilm-associated bacteria to conventional antibiotics, particularly in models of chronic *P. aeruginosa* lung infection [244].

In cancer, intercellular communication within the TME plays an analogous role in coordinating resistance [245]. Tumor cells communicate with stromal cells, immune cells, and each other through paracrine signaling (e.g., HGF, IL-6, TGF-β, Wnt ligands), exosome-mediated transfer of resistance-conferring molecules (including drug efflux transporters, microRNAs, and mutant oncoproteins), and direct cell–cell contact through gap junctions and tunneling nanotubes [246]. Disrupting these communication networks is an active area of investigation. Strategies include antibodies or small molecules targeting paracrine signaling axes (e.g., anti-HGF/c-MET therapy), exosome biogenesis inhibitors (e.g., GW4869, targeting neutral sphingomyelinase), and agents that reprogram cancer-associated fibroblasts from a resistance-promoting to a resistance-neutral or antitumor phenotype [245].

The conceptual parallel between QS in bacteria and paracrine or exosome-mediated signaling in tumors is increasingly recognized. Both systems rely on secreted molecules to coordinate population-level behaviors that promote collective survival under stress. Both generate concentration-dependent responses that scale with population density. Disrupting either system can shift the balance from a resistant, community-protected phenotype to a vulnerable, drug-susceptible state. This convergence indicates that the principles behind QS inhibition in microbiology, especially the strategy of “virulence disarmament” through disrupting cooperative communication rather than directly killing, could guide similar “resistance-disarmament” approaches in cancer treatment. In tumors, stromal-derived paracrine signals, exosome-mediated transfer of resistance factors, and fibroblast-driven remodeling of the microenvironment collectively maintain therapeutic tolerance; targeting these communication pathways has been shown to restore tumors’ sensitivity to existing therapies [247].

### 4.4. Targeting Persistence: From Bacterial Persisters to Cancer Stem Cells

Cellular dormancy and persistence, whose mechanistic basis was examined in Section 3.6, are among the most therapeutically challenging aspects of drug resistance. Dormant subpopulations, whether bacterial persisters or cancer stem-like cells, survive treatment by entering quiescent states that render them insensitive to agents that target actively proliferating cells [189,248]. Strategies to eliminate these populations focus on two complementary approaches: forcing dormant cells into a drug-susceptible proliferative state (“wake-and-kill”) or directly targeting the survival programs that sustain dormancy [249].

In bacterial infections, wake-and-kill strategies aim to reactivate persister cells, making them susceptible to conventional antibiotics. Metabolic stimulation using carbon sources such as mannitol or glucose has been shown to potentiate aminoglycoside killing of *E. coli* persisters by restoring proton motive force and active drug uptake [191]. Compounds that interfere with toxin-antitoxin (TA) modules, the stringent response ((p)ppGpp synthesis), or the SOS response are under investigation as anti-persister agents, though none have yet reached clinical use [250]. Alternative approaches include the use of acyldepsipeptide antibiotics (e.g., ADEP4), which activate the ClpP protease to degrade essential cellular proteins in dormant bacteria, effectively killing non-growing cells independently of conventional bactericidal mechanisms [191]. Bacteriophage-based strategies are also being explored, as certain engineered phages can target and lyse metabolically quiescent bacteria within biofilms [251].

In oncology, the elimination of CSCs and DTP cells has become a major research priority. Differentiation therapy, which forces CSCs out of their stem-like state and into a more differentiated, drug-sensitive phenotype, represents the oncologic counterpart of bacterial wake-and-kill strategies. All-trans retinoic acid (ATRA) in acute promyelocytic leukemia remains the most successful clinical example of this approach [173]. Small molecules targeting CSC-associated signaling pathways, including Wnt (porcupine inhibitors), Notch (gamma-secretase inhibitors), and Hedgehog (vismodegib, sonidegib), are in various stages of clinical development for solid tumors [252]. Epigenetic therapies, including HDAC inhibitors and BET bromodomain inhibitors, can disrupt the chromatin states that maintain DTP populations, potentially resensitizing residual disease to subsequent rounds of therapy [153]. Metabolic targeting of dormant cancer cells through inhibition of fatty acid oxidation or mitochondrial OxPhos, the preferred energy sources for quiescent tumor subpopulations, is also under active investigation [253].

The bidirectional translational potential is substantial. The bacterial persister field has developed rigorous quantitative frameworks for measuring persistence (minimum duration of killing assays and time-kill kinetics) and for modeling persister dynamics under fluctuating drug exposure. These methodological approaches could be adapted to quantify and predict DTP behavior in cancer. Conversely, the sophisticated single-cell profiling and lineage-tracing technologies now available in oncology could be applied to bacterial persister populations to resolve the heterogeneity within persister states at unprecedented resolution. Both fields converge on the recognition that eliminating dormant populations requires either reactivating them into a vulnerable state or directly targeting their survival dependencies, a therapeutic logic that is fundamentally shared.

### 4.5. Nanotechnology and Drug Delivery Systems

Nanotechnology-based drug delivery systems offer a versatile platform for overcoming drug resistance in both antimicrobial therapy and oncology. By enhancing drug bioavailability, enabling targeted delivery, protecting therapeutic agents from degradation or efflux, and facilitating co-delivery of multiple agents, nanocarriers address several resistance mechanisms simultaneously. The convergence of nanotechnology applications across both fields reflects the shared physical and biological barriers that limit the efficacy of conventional drugs [236,254].

In antimicrobial therapy, nanoparticle-based delivery systems have demonstrated significant potential for improving antibiotic efficacy against resistant and biofilm-associated infections. Liposomal formulations can enhance the intracellular delivery of antibiotics to bacteria residing within macrophages or epithelial cells, a strategy particularly relevant for pathogens such as *M. tuberculosis* and *Salmonella* spp., which exploit intracellular niches [254]. Polymeric nanoparticles (e.g., PLGA, chitosan-based systems) enable sustained antibiotic release at infection sites, maintaining drug concentrations above the minimum inhibitory concentration (MIC) for extended periods [255]. Metallic nanoparticles, including silver and zinc oxide nanoparticles, possess intrinsic antimicrobial properties and can act synergistically with conventional antibiotics, disrupting bacterial membranes and generating reactive oxygen species (ROS) that overwhelm microbial antioxidant defenses [256]. Nanoparticle systems have also been engineered to penetrate biofilms by exploiting their porous matrix architecture or by incorporating biofilm-dispersing enzymes on the nanoparticle surface, thereby addressing one of the most recalcitrant barriers to antibiotic efficacy [257].

In oncology, nanomedicine has progressed further toward clinical translation, with several approved formulations in routine use (reviewed in [258]). Liposomal doxorubicin (Doxil/Caelyx), nab-paclitaxel (Abraxane), and liposomal irinotecan (Onivyde) represent established examples of nanoformulations that improve pharmacokinetics, reduce systemic toxicity, and enhance tumor accumulation through the enhanced permeability and retention (EPR) effect [259]. Beyond these first-generation platforms, next-generation nanocarriers are being designed to co-deliver chemotherapeutic agents with EFIs, epigenetic modulators, or siRNAs targeting resistance genes, enabling simultaneous attack on multiple resistance mechanisms within a single delivery vehicle [260]. Stimuli-responsive nanoparticles that release their payload in response to tumor-specific conditions (low pH, elevated ROS, enzymatic activity) offer the potential for spatially and temporally controlled drug release, maximizing efficacy while minimizing off-target toxicity [261]. Antibody-conjugated nanoparticles (immunoliposomes) and aptamer-functionalized carriers further enhance targeting precision by directing delivery to specific cell-surface markers on resistant tumor cells or CSCs [262].

Several cross-disciplinary principles emerge from the parallel development of nanomedicine in both fields. Nanoparticle-mediated co-delivery of synergistic drug combinations, now a central strategy in cancer nanomedicine, could be translated to antimicrobial applications where antibiotic-EPI or antibiotic-biofilm dispersant combinations are needed. Conversely, the antimicrobial nanotechnology field has developed advanced biofilm-penetrating delivery systems that could inform the design of nanocarriers capable of traversing the dense extracellular matrix of solid tumors. Surface functionalization strategies, including ligand-targeted delivery and stimuli-responsive release, are conceptually transferable between domains. These shared technological platforms highlight nanotechnology as a particularly promising area for unified, cross-disciplinary resistance management.

### 4.6. Drug Repurposing and Host-Directed Approaches

Drug repurposing (the identification of new therapeutic applications for existing approved drugs) and host-directed approaches (strategies that modulate host biology rather than targeting the pathogen or tumor directly) represent complementary strategies for overcoming drug resistance. Both approaches offer advantages in terms of accelerated development timelines, established safety profiles, and the potential to circumvent resistance mechanisms that specifically target pathogen- or tumor-directed therapies [263,264]. Drug repurposing has gained significant traction in both antimicrobial and oncology research.

In the antimicrobial context, several non-antibiotic drugs have demonstrated activity against resistant bacteria. Statins, widely prescribed for cardiovascular disease, exhibit both immunomodulatory and direct antibacterial properties, including disruption of isoprenoid biosynthesis, interference with membrane integrity, and synergistic enhancement of antibiotic activity against *S. aureus* and other resistant pathogens [265]. The anthelmintic niclosamide has shown activity against MDR Gram-positive bacteria [266], while the anticancer agent 5-fluorouracil has been reported to inhibit bacterial biofilm formation [267]. The antifungal ciclopirox [268], the antirheumatic auranofin [269], and certain phenothiazine antipsychotics [270] have also demonstrated antimicrobial properties in preclinical studies, though clinical validation remains limited.

In oncology, drug repurposing has yielded several promising leads. The antidiabetic metformin has been extensively investigated for anticancer activity through AMPK activation and mTOR pathway inhibition, with epidemiological and preclinical data suggesting improved outcomes in several cancer types [271]. The antimalarial chloroquine and its derivative hydroxychloroquine inhibit autophagy, a survival mechanism exploited by drug-resistant tumor cells, and are being evaluated in combination with targeted therapies in clinical trials [272]. The antiparasitic ivermectin has demonstrated anticancer activity through multiple mechanisms, including inhibition of Wnt/β-catenin signaling and induction of mitochondrial dysfunction [273].

Host-directed therapies represent a conceptually distinct approach that targets the host immune response or tissue environment rather than the pathogen or tumor cell directly [274,275]. In infectious diseases, host-directed strategies aim to enhance immune clearance, reduce pathological inflammation, or modulate cellular pathways exploited by intracellular pathogens. Examples include vitamin D supplementation to enhance macrophage antimicrobial responses, metformin to augment autophagy-mediated killing of intracellular *M. tuberculosis*, and non-steroidal anti-inflammatory drugs (NSAIDs) to modulate the inflammatory milieu at infection sites [276]. Cytokine-based immunomodulation, including interferon-γ administration for refractory mycobacterial infections, represents another established host-directed strategy [277].

In oncology, host-directed approaches have achieved transformative clinical success through immune checkpoint inhibitors (anti-PD-1/PD-L1 and anti-CTLA-4 antibodies), which restore antitumor immune responses suppressed by the tumor microenvironment [278]. The extension of immunotherapy principles to infectious diseases, such as PD-1 blockade to restore exhausted T-cell responses in chronic viral infections (including HIV and hepatitis B), illustrates the bidirectional translational potential of host-directed strategies [279]. Conversely, several antibiotics and antimicrobial-derived compounds have been investigated or used as anticancer agents, illustrating the bidirectional nature of drug repurposing between infectious disease and oncology [280]. Classical antitumor antibiotics, such as doxorubicin, daunorubicin, bleomycin, mitomycin C, and dactinomycin, originate from microbial natural products and remain important components of cancer therapy [281]. Beyond these established agents, several antibacterial drugs have shown anticancer potential in preclinical or translational studies [281]. Tetracyclines, including doxycycline and minocycline, have been reported to inhibit mitochondrial translation, matrix metalloproteinases, angiogenesis, and cancer stem cell-associated phenotypes [280,282]. Macrolides and fluoroquinolones have also been explored for anticancer effects through modulation of autophagy, apoptosis, mitochondrial function, and tumor-associated inflammation [283,284]. Although most of these applications remain investigational and require further clinical validation, they reinforce the concept that antimicrobial pharmacophores may reveal vulnerabilities relevant to cancer biology and resistance management [285].

The convergence between drug repurposing and host-directed approaches across both fields highlights the value of looking beyond conventional pathogen- or tumor-specific therapeutic boundaries. Repurposed agents, including non-antibiotic drugs with antimicrobial activity and antimicrobial-derived compounds with anticancer potential, can modulate shared biological processes such as autophagy, mTOR signaling, mitochondrial function, oxidative stress responses, and microenvironmental inflammation. These overlapping vulnerabilities may be exploited to address resistance mechanisms common to both microbial and cancer systems. In parallel, host-directed strategies, by targeting the host response or tissue environment rather than the evolving pathogen or tumor cell directly, may be less susceptible to the genetic and phenotypic escape mechanisms that limit conventional therapies. Together, these approaches reinforce drug repurposing and host-directed intervention as complementary components of cross-disciplinary resistance management [279,286].

### 4.7. Disrupting Protective Microenvironments: Biofilm and Tumor Niche Targeting

The protective microenvironments discussed in Section 3.7, whether bacterial biofilms or the TME, represent significant barriers to effective therapy. Strategies that disrupt these protective niches, rather than targeting resistant cells directly, can enhance drug penetration, restore immune access, and resensitize otherwise tolerant populations to conventional therapies. This subsection examines emerging approaches to microenvironment disruption in both domains and identifies translational principles shared between them.

In infectious diseases, biofilm disruption strategies target the structural integrity and signaling networks that maintain the biofilm community. Enzymatic degradation of the EPS matrix using DNase I (which degrades extracellular DNA scaffolding), dispersin B (which hydrolyzes poly-N-acetylglucosamine), and alginate lyase (which degrades the alginate matrix of *P. aeruginosa* biofilms) has shown efficacy in preclinical biofilm models [287]. Small molecules that trigger biofilm dispersal by modulating intracellular signaling, particularly those that reduce cyclic-di-GMP levels (a key second messenger that promotes the biofilm phenotype), are under active development [288]. Anti-adhesion strategies that prevent initial biofilm attachment, including mannosides that block FimH-mediated adhesion of uropathogenic *E. coli* to bladder epithelium, represent a prophylactic approach to biofilm prevention [289]. Bacteriophage therapy offers an additional dimension, as many phages produce EPS-degrading depolymerases that enable penetration into established biofilms, and engineered phages can deliver biofilm-dispersing enzymes directly to the infection site [290].

In oncology, TME-targeted strategies aim to normalize or disrupt the stromal, vascular, and immune components that shield tumor cells from therapy. Vascular normalization using anti-VEGF agents (e.g., bevacizumab) transiently restores functional tumor vasculature, improving drug delivery and reducing hypoxia-driven resistance [291]. ECM-targeting approaches include the use of hyaluronidase (PEGPH20) to degrade hyaluronan-rich stroma in pancreatic cancer, though clinical results have been mixed, and collagenase-based strategies to reduce ECM density and improve drug penetration [292,293]. Targeting cancer-associated fibroblasts (CAFs), which produce pro-survival growth factors and physically exclude therapeutic agents, is being explored through FAP-targeted therapies and agents that reprogram CAF phenotypes [294]. Immunomodulatory strategies that convert the immunosuppressive TME into an immune-permissive environment, including colony-stimulating factor 1 receptor (CSF1R) inhibitors to repolarize tumor-associated macrophages and combination approaches with immune checkpoint inhibitors, aim to restore effective antitumor immunity within the niche [295].

The translational parallels between biofilm disruption and TME modulation are substantial. Both approaches share a common therapeutic logic: rather than targeting resistant cells directly, they dismantle the protective infrastructure that sustains resistance. Enzymatic matrix degradation (DNase for biofilm EPS; hyaluronidase for tumor ECM) represents a directly analogous strategy across domains. Signaling disruption (QS inhibition in biofilms; paracrine signaling blockade in the TME) follows the same principle of interrupting the communication networks that maintain community-level protection. Immune restoration, whether by exposing biofilm bacteria to phagocytic clearance or by reprogramming the immunosuppressive TME, reflects a convergent recognition that microenvironmental immune evasion is a shared obstacle to effective therapy. These parallels support the development of platform technologies, particularly nanomedicine-based delivery systems capable of co-delivering matrix-disrupting enzymes alongside antimicrobial or anticancer agents, that could be adapted across both clinical contexts.

## 5. Challenges and Limitations of Cross-Domain Translation

While the mechanistic parallels between microbial and cancer drug resistance provide a compelling rationale for cross-disciplinary innovation, several fundamental biological, pharmacological, regulatory, and economic challenges must be acknowledged. Recognizing these limitations is essential for setting realistic expectations and identifying the specific conditions under which cross-domain translation is most likely to succeed.

### 5.1. Fundamental Biological Differences

The most significant barrier to direct translation lies in the fundamental biological divergence between prokaryotic microorganisms and eukaryotic tumor cells. Bacteria and cancer cells differ in genome organization, cellular architecture, replication mechanisms, and the complexity of their regulatory networks. Bacterial resistance frequently involves horizontally transferable genetic elements, such as plasmids, integrons, and transposons, that enable rapid population-level dissemination of resistance determinants, a mechanism without a direct counterpart in cancer, where resistance is primarily driven by clonal evolution within a single host.

Furthermore, the relatively compact and modular nature of bacterial genomes often allows resistance phenotypes to be attributed to discrete, well-characterized genetic determinants. In contrast, cancer drug resistance typically involves polygenic, context-dependent interactions across multiple signaling pathways, epigenetic states, and microenvironmental influences that are far more difficult to disentangle. Consequently, therapeutic strategies targeting a single resistance mechanism may be more effective in bacterial infections than in cancer, where redundancy and compensatory pathway activation frequently limit the durability of targeted interventions.

### 5.2. Differences in Evolutionary Timescales and Population Dynamics

Although both microbial and tumor populations evolve under selective pressure, their evolutionary timescales and population dynamics differ substantially. Bacterial populations can undergo thousands of generations within days, enabling rapid emergence and fixation of resistance traits. Tumor cell populations, while genetically unstable, typically evolve over longer periods, with additional layers of complexity introduced by immune surveillance, tissue architecture, and microenvironmental heterogeneity.

These differences have important implications for resistance-management strategies. Treatment schedules, dosing intervals, and drug-cycling approaches optimized for bacterial infection kinetics may not translate directly to the slower and more complex dynamics of tumor evolution. Accordingly, mathematical and evolutionary models developed in one domain require careful recalibration before application to the other.

### 5.3. Pharmacological and Pharmacokinetic Barriers

Drug development in infectious diseases and oncology operates under fundamentally different pharmacological constraints. Antimicrobial agents must achieve bactericidal or bacteriostatic concentrations at the site of infection within defined pharmacokinetic–pharmacodynamic (PK–PD) windows, often over relatively short treatment durations. In contrast, anticancer therapies are frequently administered over prolonged periods and operate within narrow therapeutic indices, where cumulative toxicity, drug–drug interactions, and pharmacogenomic variability significantly influence treatment outcomes.

The challenge of selectivity also differs markedly. Antibiotics typically exploit prokaryote-specific targets, such as cell wall synthesis, bacterial ribosomes, or folate metabolism, that are absent in host cells. Anticancer agents, however, target eukaryotic pathways shared between malignant and normal cells, inherently limiting therapeutic selectivity. As a result, strategies such as efflux pump inhibition, metabolic modulation, or epigenetic targeting cannot be directly translated across domains without careful evaluation of toxicity, specificity, and therapeutic index.

### 5.4. Regulatory and Clinical Trial Asymmetries

The regulatory and economic landscapes governing antimicrobial and anticancer drug development differ considerably. Oncology has benefited from accelerated approval pathways, breakthrough therapy designations, companion diagnostic integration, and orphan drug incentives, which have facilitated rapid clinical translation. In contrast, antimicrobial development continues to face structural economic challenges. Despite initiatives such as the Generating Antibiotic Incentives Now (GAIN) Act and European programs under the Innovative Medicines Initiative (IMI), new antibiotics are often reserved through stewardship programs, limiting commercial returns and discouraging sustained industry investment.

Clinical trial design also diverges between fields. Oncology increasingly employs biomarker-driven patient stratification and adaptive trial designs to evaluate combination and sequential therapies. In contrast, antimicrobial trials frequently rely on non-inferiority designs, which are less suited to demonstrating the added value of resistance-targeting strategies. These asymmetries constrain the bidirectional translation of therapeutic innovations.

### 5.5. Risk of Oversimplification

A broader conceptual limitation lies in the risk of oversimplifying cross-domain comparisons. While identifying shared resistance mechanisms is valuable, superficial analogies may obscure important biological distinctions. For example, bacterial efflux pumps and cancer-associated ABC transporters share functional similarities but differ in substrate specificity, regulatory control, membrane context, and physiological role. Likewise, bacterial persister cells and cancer DTPcells both exhibit reversible dormancy, yet the molecular pathways governing these states are distinct.

Therefore, translational conclusions must be grounded in rigorous validation at the molecular, cellular, and in vivo levels. Functional analogy should not be conflated with mechanistic identity.

### 5.6. Limitations of This Review: Scope, Methodology, and Interpretive Boundaries

In addition to the biological, pharmacological, and translational challenges discussed above, this narrative review has several methodological and scope-related limitations that should be explicitly acknowledged. As a narrative review, this work is subject to inherent methodological limitations, including potential selection bias in the literature coverage, the absence of quantitative synthesis, and reliance on the author’s interpretive framework for thematic organization. Notably, narrative reviews cannot provide the same level of evidence as systematic reviews or meta-analyses.

The intentionally broad scope of this review, designed to capture cross-domain parallels, necessarily limits the depth of discussion of individual mechanisms or therapeutic strategies. Emerging areas, such as artificial intelligence for resistance prediction, phage therapy, and microbiome-driven modulation of drug metabolism, are acknowledged but not comprehensively addressed. Future work that incorporates systematic methodology and quantitative cross-domain analyses would strengthen the evidence base for the proposed translational framework. Specific research priorities and policy recommendations are outlined in Section 6.

## 6. Perspectives and Recommendations

The cross-disciplinary framework developed in this review highlights both the promise and the complexity of translating insights from microbiology into oncology. While the preceding sections have outlined shared mechanisms, therapeutic strategies, and the challenges that constrain translation, the full potential of this convergent perspective remains largely unrealized. This section outlines specific research priorities, policy directions, and structural recommendations to accelerate progress toward integrated resistance management across both domains.

### 6.1. Research Priorities

#### 6.1.1. Unified Molecular Characterization of Resistance

A fundamental gap in cross-domain resistance research is the absence of standardized molecular frameworks that enable direct comparison of resistance mechanisms across biological systems. While individual mechanisms (efflux, target modification, persistence) have been characterized in depth within their respective fields, systematic side-by-side molecular analyses remain rare. Future research should prioritize comparative studies that examine orthologous resistance pathways, such as ABC transporter families, at the structural, functional, and regulatory levels across bacterial and cancer contexts. High-resolution structural biology approaches (cryo-EM, X-ray crystallography) applied to both bacterial and eukaryotic efflux transporters could identify shared druggable sites and inform the design of broad-spectrum inhibitors with cross-domain applicability [296]. Similarly, comparative epigenomic profiling of bacterial persister cells and cancer DTP populations could reveal conserved chromatin or methylation signatures that govern entry into and exit from dormant states, providing potential therapeutic targets relevant to both fields [297].

#### 6.1.2. Single-Cell and Spatial Technologies

The advent of single-cell transcriptomics, proteomics, and spatial biology offers transformative opportunities for understanding resistance heterogeneity in both microbial and tumor populations. In oncology, scRNA-seq and spatial transcriptomics are already being applied to map intratumoral heterogeneity and identify rare resistant subpopulations, including DTP cells and CSCs, at unprecedented resolution [298]. The application of analogous technologies to bacterial populations, while technically more challenging due to the lower RNA content per cell, is advancing rapidly and could enable the identification of persister-specific transcriptional programs, heteroresistant subpopulations, and spatially defined resistance niches within biofilms [298]. Integrating single-cell datasets across both domains could reveal conserved gene expression signatures of drug tolerance that transcend the prokaryote-eukaryote divide, potentially identifying universal biomarkers of therapeutic vulnerability.

#### 6.1.3. Computational and Artificial Intelligence Approaches

Artificial intelligence (AI) and machine learning (ML) are increasingly being deployed to predict resistance evolution, optimize combination therapy design, and identify drug repurposing candidates. In antimicrobial research, ML models trained on whole-genome sequencing data can accurately predict resistance phenotypes across several bacterial species and antibiotic classes [299]. Recent studies have further underscored the value of integrating AI with genome sequencing and antimicrobial susceptibility data to improve resistance detection, surveillance, and prediction, while also highlighting the need for external validation, explainable AI, and standardized datasets before clinical implementation [300,301]. In oncology, AI-driven analysis of tumor genomic and transcriptomic profiles is being used to predict treatment response and identify resistance-associated molecular signatures [302]. Recent oncology-focused AI frameworks and drug-resistance prediction tools have also begun integrating pharmacogenomic, molecular marker, and drug-sensitivity datasets to support biomarker discovery, patient stratification, and prediction of resistance-associated therapeutic failure [303,304]. A significant opportunity lies in developing cross-domain AI platforms that integrate resistance data from both microbial and cancer systems, leveraging the larger, more standardized datasets available in antimicrobial surveillance (e.g., GLASS, EARS-Net) to train models that can generate transferable predictions of cancer resistance dynamics. Evolutionary modeling, including fitness landscape analysis and adaptive dynamics simulations, represents another area where computational approaches developed in microbial ecology could be applied to tumor evolution, and vice versa [305]. Reinforcement learning algorithms that optimize drug dosing and sequencing strategies in real time are being explored in both fields and could benefit from shared methodological development [306]. Recent reinforcement learning and deep reinforcement learning models have extended this concept by optimizing adaptive cancer therapy schedules and personalized chemotherapy regimens, providing a computational framework for dynamically suppressing resistant populations while limiting toxicity [307,308].

#### 6.1.4. In Vivo and Clinical Validation

To date, most cross-domain mechanistic comparisons have been conducted in vitro. Translating these insights into clinical impact requires investment in in vivo models that capture the complexity of resistance across both domains. For antimicrobial research, this includes chronic infection models that incorporate biofilm dynamics, immune responses, and pharmacokinetic variability. For oncology, patient-derived xenografts (PDXs), organoid systems, and genetically engineered mouse models that recapitulate intratumoral heterogeneity and microenvironmental complexity are essential. Importantly, clinical trials designed to test cross-domain therapeutic concepts (e.g., combination regimens inspired by antimicrobial PK-PD principles, or persistence-targeting strategies adapted from bacterial persister research) should be prioritized. Adaptive trial designs that allow real-time modification of treatment arms based on emerging resistance data could accelerate the clinical evaluation of these strategies in both infectious disease and oncology settings [309,310].

#### 6.1.5. Early Detection of Resistance During Drug Development

Early identification of resistance liabilities during drug development is essential to the design of more durable antimicrobial and anticancer therapies [311]. In antimicrobial research, serial passage experiments, mutant prevention concentration assays, time-kill studies, and experimental evolution platforms help determine how rapidly resistance develops under drug pressure and can predict potential mutation pathways before clinical deployment [312]. Whole-genome sequencing of resistant isolates from preclinical testing can further delineate resistance determinants, including target-site mutations, efflux activation, permeability changes, and compensatory adaptations. These approaches can inform chemical optimization, combination design, and dosing strategies to minimize the selection of resistant mutants [313,314].

In oncology, resistance-detection strategies rely on prolonged drug exposure in molecularly annotated cancer cell lines, patient-derived organoids that preserve clonal heterogeneity, patient-derived xenografts that enable in vivo tracking of acquired resistance under therapeutic pressure, and single-cell-integrated functional genomics platforms that resolve resistance-associated transcriptional states prior to clinical translation (reviewed in [315]). Genomic, transcriptomic, epigenomic, and proteomic profiling of drug-tolerant or resistant subpopulations can reveal early adaptive states, bypass signaling pathways, and biomarkers of reduced drug sensitivity. Single-cell and spatial technologies are particularly valuable for detecting rare pre-existing resistant clones or DTP populations that bulk profiling may miss. Integrating these experimental approaches with pharmacokinetic–pharmacodynamic modeling, evolutionary forecasting, and AI-driven prediction tools could help identify resistance risks earlier in the development pipeline and inform rational combination or sequencing strategies [110,316].

A cross-disciplinary framework for early resistance detection would therefore benefit both fields. Antimicrobial drug development can provide robust experimental evolution and mutant-selection paradigms, while oncology offers advanced single-cell, organoid, and biomarker-driven platforms. Incorporating resistance-risk assessment into routine preclinical drug development could improve candidate prioritization, inform trial design, and reduce the likelihood of rapid therapeutic failure after clinical introduction.

### 6.2. Policy and Funding Recommendations

Current funding structures in most countries maintain a rigid separation between infectious disease and oncology research portfolios. National and international funding agencies, including the National Institutes of Health (NIH), the European Research Council (ERC), and the Wellcome Trust, should consider establishing dedicated cross-disciplinary funding streams that incentivize collaborative research on shared resistance mechanisms. Joint calls for proposals that require co-investigators from both microbiology and oncology, or that mandate the inclusion of cross-domain translational aims, could catalyze the formation of new research networks and accelerate knowledge transfer.

Regulatory frameworks should evolve to accommodate cross-domain therapeutic development. The success of orphan drug incentives and accelerated approval pathways in oncology offers a model that could be adapted for antimicrobial resistance, particularly for agents targeting shared resistance mechanisms (e.g., efflux inhibitors, anti-persistence compounds) that may have dual-domain applicability. Regulatory agencies, including the FDA and EMA, could explore developing cross-indication approval pathways for agents with demonstrated activity against conserved resistance targets in both microbial and cancer systems, provided adequate safety and efficacy data are available for each indication. The integration of resistance biomarkers into regulatory decision-making, already advanced in oncology through companion diagnostics, should be expanded in antimicrobial therapy to enable precision approaches to resistance management.

Antimicrobial resistance surveillance is well established at the global level through networks such as GLASS and EARS-Net, as well as national programs, including the CDC Antibiotic Resistance Threats Reports. Cancer drug resistance, by contrast, lacks an equivalent systematic surveillance infrastructure. The development of cancer resistance registries that capture treatment-resistance phenotypes, molecular resistance mechanisms, and clinical outcomes at the population level would enable epidemiological analyses comparable to those that have transformed our understanding of AMR. Where possible, integrating resistance surveillance data across infectious disease and oncology, for example, through shared bioinformatics platforms and standardized resistance ontologies, could help identify cross-domain resistance trends and inform both clinical practice and drug development priorities.

### 6.3. Educational and Structural Recommendations

The next generation of resistance researchers and clinicians would benefit from training programs that integrate microbiology, oncology, evolutionary biology, and pharmacology. Graduate curricula that include comparative resistance biology, cross-domain pharmacology, and translational research methodology could cultivate a cadre of investigators prepared to work across traditional disciplinary boundaries. Joint seminars, laboratory rotations, and co-mentorship arrangements between microbiology and oncology departments are practical steps toward this goal.

Effective cross-disciplinary communication is hindered by terminological inconsistencies between fields. Concepts such as “persistence,” “tolerance,” “heteroresistance,” and “adaptive resistance” carry different connotations and operational definitions in microbiology and oncology. An expert-driven initiative to harmonize resistance terminology across domains, analogous to the standardization efforts that have improved consistency in antimicrobial susceptibility testing (e.g., CLSI and EUCAST guidelines), would facilitate clearer communication, more rigorous cross-domain comparisons, and greater reproducibility in translational research.

Establishing international, cross-disciplinary research consortia focused on resistance biology could provide the organizational infrastructure needed to sustain long-term collaboration. Models such as the European Joint Program on Antimicrobial Resistance (EJP AMR) and the Cancer Grand Challenges initiative demonstrate the feasibility and impact of large-scale collaborative research programs. A comparable initiative that explicitly links AMR and cancer drug resistance research could coordinate multi-site studies, share datasets and biobanks, and provide a platform for the rapid dissemination of cross-domain findings.

### 6.4. Toward an Integrated Resistance Management Framework

Taken together, these recommendations support a central proposition: drug resistance should be reconceptualized as a unified biological challenge requiring integrated scientific, clinical, regulatory, and policy responses. The current siloed approach, in which microbiology and oncology operate largely independently, is increasingly insufficient to address shared evolutionary pressures. An integrated resistance management framework, grounded in the mechanistic parallels outlined in this review and supported by coordinated structural reforms, has the potential to accelerate therapeutic innovation, improve clinical outcomes, and reduce the global burden of drug-resistant disease across both domains.

## 7. Conclusions

Drug resistance remains one of the most consequential challenges in contemporary medicine, driving treatment failure, disease recurrence, and mortality across both microbial infections and cancer. This narrative review has examined drug resistance as a convergent evolutionary phenomenon, demonstrating that microbial pathogens and tumor cells, despite their fundamental biological differences, employ strikingly similar strategies to survive pharmacological pressure. Six core resistance mechanisms shared across both domains were identified and analyzed: efflux-mediated drug export, target modification and mutation, enzymatic drug inactivation, epigenetic and regulatory plasticity, metabolic reprogramming, and the exploitation of protective microenvironments. These mechanisms are further reinforced by phenotypic heterogeneity, including bacterial persister cells and cancer stem-like cells, which generate drug-tolerant subpopulations that resist eradication and serve as reservoirs for disease relapse. Together, these parallels establish drug resistance as a universal biological process rooted in adaptability and selective pressure rather than a domain-specific clinical problem. The recognition of these shared mechanisms opens significant translational opportunities. Combination therapy, efflux inhibition, QS and intercellular signaling disruption, persistence-targeting strategies, nanotechnology-enabled drug delivery, drug repurposing, host-directed approaches, and microenvironment-disrupting interventions were identified as cross-disciplinary strategies with the potential to prevent or delay resistance, or overcome it, in both fields. Several of these approaches have already demonstrated preclinical promise, and their further development stands to benefit from bidirectional knowledge transfer between microbiology and oncology. At the same time, this review has acknowledged the substantial challenges that constrain cross-domain translation. Fundamental biological differences between prokaryotic and eukaryotic systems, divergent evolutionary timescales and population dynamics, pharmacological and pharmacokinetic barriers, regulatory asymmetries, and the risk of oversimplifying mechanistic analogies all impose boundaries on the transferability of resistance insights and therapeutic strategies. These limitations do not negate the value of cross-disciplinary thinking but rather define the conditions under which such translation is most likely to succeed: when supported by rigorous molecular validation, appropriate in vivo modeling, and careful attention to domain-specific biological context. Looking forward, the perspectives and recommendations outlined in this review, spanning unified molecular characterization, single-cell and computational technologies, cross-disciplinary funding and regulatory reform, surveillance integration, and interdisciplinary training, provide a roadmap for advancing the integrated study and management of drug resistance. The central proposition of this review is that drug resistance should be reconceptualized as a unified biological challenge that transcends traditional disciplinary boundaries. By bridging the conceptual and methodological divide between microbiology and oncology, the scientific community can develop more durable, broadly applicable therapeutic strategies and ultimately improve outcomes for patients facing drug-resistant infections and cancers alike.

## Figures and Tables

**Table 2:** Major Mechanisms of Cancer Drug Resistance.

Mechanism	Description/Examples	Refs.
Drug efflux via ATP-binding cassette (ABC) transporters	Overexpression of ABC transporters (e.g., P-glycoprotein/MDR1, BCRP, MRP family) reduces intracellular drug concentrations and drives multidrug resistance.	[24,66,67]
Altered drug targets	Mutations, alternative splicing, or post-translational modifications alter drug–target interactions, diminishing drug binding or efficacy (e.g., EGFR and BCR-ABL variants).	[68,69]
Enhanced DNA repair and apoptosis evasion	Upregulation of DNA repair pathways and defects in apoptosis allow tumor cells to survive genotoxic stress from chemotherapy or radiation.	[24,70]
Tumor microenvironment-mediated Protection	Interactions with cancer-associated fibroblasts, immune cells, hypoxia, and extracellular matrix components create protective niches that promote survival and resistance.	[23,71]
Cancer stem cells (CSCs) and cellular plasticity	CSCs and highly plastic cell states enable persistence, relapse, and adaptation under therapeutic pressure, driven by enhanced repair and stemness-associated programs.	[23,72]
Activation of alternative signaling pathways	Compensatory activation of pathways (PI3K/Akt/mTOR, MAPK, EGFR, Wnt/β-catenin, NF-κB) bypasses inhibited drug targets and sustains proliferation.	[60,70,73]
Epithelial-to-mesenchymal transition (EMT) and metabolic reprogramming	EMT-driven transcriptional changes and rewired metabolic states promote drug tolerance, invasion, metastasis, and apoptotic resistance.	[68,74]

**Table 3:** Shared Mechanistic Principles of Drug Resistance in Microbial Infections and Cancer ^1^.

Mechanism	Microbial Context	Cancer Context
Efflux-Mediated Drug Export	Multidrug efflux pumps (e.g., AcrAB-TolC, MexAB-OprM) reduce intracellular antibiotic accumulation.	ABC transporters (e.g., P-glycoprotein/MDR1, BCRP, MRP1) lower intracellular concentrations of chemotherapeutic agents.
Target Modification/ Mutation	Point mutations in antibiotic-binding sites (e.g., rpoB, gyrA), altered PBPs, ribosomal modifications reduce drug binding.	Mutations or structural alterations in therapeutic targets (e.g., EGFR, BCR-ABL, KRAS, ALK) impair drug–target interaction.
Drug Inactivation	Enzymatic degradation or modification of antibiotics (β-lactamases, ESBLs, carbapenemases, aminoglycoside-modifying enzymes).	Detoxification enzymes (e.g., glutathione S-transferases and ALDHs) neutralize chemotherapeutic agents.
Epigenetic and Regulatory Changes	Phase variation, transcriptional regulators (MarA, SoxS, RamA), small RNAs, quorum sensing, and biofilm-associated regulatory circuits dynamically reprogram gene expression to promote tolerance.	DNA methylation, histone modification, chromatin remodeling, EMT-associated transcriptional programs, and non-genetic plasticity drive drug tolerance and phenotype switching.
Enhanced DNA Repair	SOS response, RecA, and error-prone polymerases repair antibiotic-induced DNA damage.	Upregulation of DNA repair pathways (e.g., PARP, BRCA1/2, ATM/ATR) confers resistance to genotoxic agents and radiotherapy.
Cellular Dormancy/ Persistence	Persister cells maintain a non-dividing, antibiotic-tolerant phenotype without genetic mutation.	Dormant and slow-cycling tumor cells, including cancer stem cells, survive therapy and drive recurrence.
Microenvironmental Protection	Biofilm extracellular matrix limits drug penetration, facilitates quorum-sensing-driven tolerance, and provides a protective niche.	Tumor microenvironment (CAF-rich stroma, hypoxia, ECM remodeling, immunosuppression) reduces drug penetration and promotes adaptive survival.
Metabolic Reprogramming	Stress-induced metabolic shifts (e.g., altered respiration, nutrient scavenging) support antibiotic tolerance.	Warburg effect, mitochondrial rewiring, redox remodeling, and metabolic plasticity enhance resistance to targeted therapy and chemotherapy.

^1^ Data obtained from [62,85,86].

**Table 4:** Representative cross-disciplinary therapeutic strategies for overcoming drug resistance in microbial infections and cancer.

Resistance Mechanism	Microbial Strategy	Cancer Strategy	Therapeutic Approaches	Translational Opportunities	Refs.
Combination therapy and multi-target inhibition	Antibiotic combinations (e.g., β-lactam + β-lactamase inhibitor); synergistic targeting of distinct pathways	Chemotherapy combinations; targeted therapy combinations (e.g., BRAF + MEK inhibitors); chemo-immunotherapy	Rational drug combinations; adaptive therapy; collateral sensitivity-based strategies	Evolution-informed treatment design; suppression of resistance emergence through multi-target pressure	[80,219,220]
Efflux-mediated drug export	Multidrug efflux pumps (e.g., AcrAB-TolC, NorA) reduce intracellular antibiotic concentration	Overexpression of ABC transporters (e.g., ABCB1/P-gp, ABCC1, ABCG2)	Efflux pump inhibitors (EPIs); nanoparticle-based drug delivery; drug modification	Development of broad-spectrum EPIs; nanocarriers to bypass efflux	[102,221]
Target modification and mutation	Mutations in drug targets (e.g., DNA gyrase, PBPs); rRNA methylation	Mutations in EGFR, BCR-ABL, BRAF; target amplification	Next-generation inhibitors; combination therapy; adaptive dosing	Design of mutation-resistant drugs; predictive resistance modeling	[69,80,113]
Drug inactivation and detoxification	β-lactamases; aminoglycoside-modifying enzymes	GSTs, UGTs; microbiota-mediated drug metabolism	Enzyme inhibitors; drug modification; microbiome-targeted strategies	Targeting detoxification pathways; microbiota modulation	[85,86,142]
Metabolic reprogramming	Altered metabolic flux; stress-adaptive metabolism	Warburg effect; redox adaptation; metabolic plasticity	Metabolic inhibitors; redox modulators; combination therapy	Targeting shared metabolic vulnerabilities	[175,222,223]
Epigenetic and regulatory changes	DNA methylation; phase variation; non-coding RNAs	DNA methylation; histone modification; chromatin remodeling	Epigenetic drugs (DNMT, HDAC inhibitors); transcriptional targeting	Reversal of drug-tolerant states; targeting plasticity	[153,154,157]
Cellular dormancy and persistence	Persister cells; transient antibiotic tolerance	Cancer stem cells; drug-tolerant persister (DTP) cells	Dormancy-targeting strategies; sequential therapy	Elimination of persistent reservoirs; relapse prevention	[68,166]
Protective microenvironments	Biofilms; extracellular matrix barriers	Tumor microenvironment (hypoxia, CAFs, ECM, immune evasion)	Biofilm disruptors; ECM-targeting; immunotherapy; hypoxia-targeting	Targeting protective niches; improving drug penetration	[45,68,158]

Abbreviations: ABC, ATP-binding cassette; ABCB1, ATP-binding cassette subfamily B member 1; ABCC1, ATP-binding cassette subfamily C member 1; ABCG2, ATP-binding cassette subfamily G member 2; BCR-ABL, breakpoint cluster region–Abelson tyrosine kinase fusion; BRAF, B-Raf proto-oncogene, serine/threonine kinase; CAFs, cancer-associated fibroblasts; DNMT, DNA methyltransferase; DTP, drug-tolerant persister; ECM, extracellular matrix; EGFR, epidermal growth factor receptor; EPI, efflux pump inhibitor; GST, glutathione S-transferase; HDAC, histone deacetylase; MEK, mitogen-activated protein kinase kinase; PBPs, penicillin-binding proteins; P-gp, P-glycoprotein; rRNA, ribosomal RNA; UGT, UDP-glucuronosyltransferase.

## Data Availability

No new data were created or analyzed in this study. Data sharing is not applicable to this article.
